# A Novel, Unbiased Analysis Approach for Investigating Population Dynamics: A Case Study on *Calanus finmarchicus* and Its Decline in the North Sea

**DOI:** 10.1371/journal.pone.0158230

**Published:** 2016-07-01

**Authors:** Danny J. Papworth, Simone Marini, Alessandra Conversi

**Affiliations:** 1 Faculty of Science and Technology, School of Marine Science and Engineering, Plymouth University, Plymouth, Devon, PL4 8AA, United Kingdom; 2 ISMAR–Marine Sciences Institute in La Spezia, CNR–National Research Council of Italy, Forte Santa Teresa, Loc. Pozzuolo, 19032, Lerici, SP, Italy; 3 Marine Institute, Plymouth University, Plymouth, Devon, PL4 8AA, United Kingdom; Institute of Marine Research, NORWAY

## Abstract

Marine populations are controlled by a series of drivers, pertaining to both the physical environment and the biological environment (trophic predator-prey interactions). There is heated debate over drivers, especially when trying to understand the causes of major ecosystem events termed regime shifts. In this work, we have researched and developed a novel methodology based on Genetic Programming (GP) for distinguishing which drivers can influence species abundance. This methodology benefits of having no *a priori* assumptions either on the ecological parameters used or on the underlying mathematical relationships among them. We have validated this methodology applying it to the North Sea pelagic ecosystem. We use the target species *Calanus finmarchicus*, a key copepod in temperate and subarctic ecosystems, along with 86 biological, hydrographical and climatic time series, ranging from local water nutrients and fish predation, to large scale climate pressure patterns. The chosen study area is the central North Sea, from 1972 to 2011, during which period there was an ecological regime shift. The GP based analysis identified 3 likely drivers of *C*. *finmarchicus* abundance, which highlights the importance of considering *both* physical and trophic drivers: temperature, North Sea circulation (net flow into the North Atlantic), and predation (herring). No large scale climate patterns were selected, suggesting that when there is availability of both data types, local drivers are more important. The results produced by the GP based procedure are consistent with the literature published to date, and validate the use of GP for interpreting species dynamics. We propose that this methodology holds promises for the highly non-linear field of ecology.

## Introduction

Ecosystems dynamics are an integrated response of the ecosystem’s biological components (species/groups) to drivers, which act independently, synergistically or even antagonistically [1, 2]. These drivers are defined as any natural or human-induced factor that directly or indirectly causes a change in an ecosystem or population [3].

A long, unsolved ecological question is whether top-down trophic drivers (i.e. predation) or bottom-up drivers (often intended as climate, hydrography, food) control populations and ecosystem states [4]. With regard to these terms, Conversi *et al* [2] note that the usage of climate variables as bottom-up, although widely used, may not be entirely correct for the marine environment: in fact, climate related variables, such as temperature and other physical factors are likely to simultaneously affect several trophic levels in the marine food chain [5], or may skip some levels [6], hence are not operating in a strictly “bottom-up” manner. Hence, in this article we use Conversi *et al* [2] definitions of drivers: “*physical drivers*”, which are related to the physical environment, and “*trophic*” (or “*biological*”) drivers, which are related to predator-prey interactions. Only the latter are subdivided in bottom-up and top-down drivers.

Several studies have suggested that physical processes such as climate-induced temperature or circulation changes are the main drivers for planktonic populations [7–23], whilst others suggest that human induced top-down changes, such as overfishing, which leads to changes in trophic level structure and to trophic cascading, are the main drivers [24–31]. These theories are then confronted by the idea of synergistic relationships, where a combination of drivers control a population and a change in the population depends on how drivers affect each other and/or on the resilience of the ecosystem [1, 2, 4–6, 32–43], and the debate is still open.

In this work, we address the question of the drivers of planktonic populations using a novel, holistic approach, in which multiple, potential drivers are analysed without assumptions on either their relative importance or the mathematical relationship between them and the target species.

These potential drivers are analysed using a symbolic regression methodology based on genetic programming (GP). GP [44–46] is a domain-independent evolutionary computation methodology, capable of generating solutions to a given problem without any strong *a-priori* knowledge or assumption on the problems solution (see S1 File for details). GP has been used successfully in a wide range of applications [46], such as robotics [47, 48], physics [49], stock market analysis [50], and medicine [51]. Its use for understanding environmental patterns and drivers is very recent, for example, investigating the causes of copepod variability in the English Channel [52], predicting harmful algal blooms [53], predicting early warning of cyanobacterial blooms in freshwater ecosystems [54], and the energy output of wind farms based on weather data [55]. As well as predictions, genetic algorithms have been used to parameterise coupled biological–physical copepod population-dynamics computations [56]. In particular, the GP-based approaches in [54] have been compared with other methodologies for multivariate function fitting and knowledge discovery and have shown superior quantitative performance.

The proposed GP-based analysis is conceptually opposite to the mechanistic approaches where modelling is based on *a-priori* knowledge and the model parametrizations are based on literature values and laboratory experiments (e.g., [57, 58]). Quite the reverse, the symbolic regression methodology based on GP is capable of extracting mathematical models *from* the data, whose interpretation can provide useful information on the investigated context. These properties make it worth investigating how this methodology can be applied for analysing ecosystems, in particular for detecting variables that can be relevant for (i.e., explain ecosystem variability of) a target variable, and for identifying the mathematical models governing them [45, 59, 60]. The GP approach can be of particular service in the analysis of population dynamics, since species may respond to environmental change in a nonlinear manner (as in the case of ecological regime shifts), and the usual linear analyses may not be appropriate.

In this work, we aim to validate a novel methodology based on GP at distinguishing which drivers can influence species abundance. We use the target species *Calanus finmarchicus* in the North Sea, a key copepod in temperate and subarctic ecosystems, along with 86 biological, hydrographical and climatic time series, ranging from local water nutrients and fish predation, to large scale climate pressure patterns.

### *Calanus finmarchicus* in the North Sea

The North Sea is situated on the continental shelf of northwest Europe. It opens into the Norwegian Sea and the Atlantic Ocean to the north, into the Atlantic Ocean to the southwest via the English Channel, and into the Baltic Sea to the east through the Skagerrak Strait. The North Sea is often divided into the shallow southern North Sea (<50 m depth), the central North Sea, the northern North Sea (~200 m depth), the Norwegian Trench (up to 700 metres deep) and the Skagerrak. In the North Sea, North Atlantic water mixes with freshwater runoff and river discharges in a predominantly anti-clockwise circulation. Smaller currents move southwards along the east coast of the UK and northwards along the continental western European coast. Shallow areas of the North Sea (<30 m) are generally fully mixed by tidal action, whilst deeper areas have a surface mixed layer (upper 30 m) which is usually mixed by wind action [61, 62].

The North Sea is an area of high importance for commercial fisheries and wildlife, and once provided 5% of the global fish harvest [41, 63]. However, the North Sea is under increasing pressure from the effects of climate change and associated temperature increase [64] and human populations; with increased coastal development, pollution, nutrient input, maritime transport, as well as food and energy demands with around 30 different commercial fish stocks still being exploited, and interests from gas, oil and renewable energy industries [61, 62]. It has been an area of focus and debate on the drivers of pelagic populations due to a series of abrupt ecosystem transitions, termed regime shifts [2, 65, 66]. These events affected the entire North Sea ecosystem, however, the exact timings and causes are inconclusive, with physical and trophic possible drivers, as well as synergistic combinations, being identified using a variety of methods [21, 32, 66–76].

Copepods constitute a key trophic group, transferring energy from phytoplankton to higher trophic levels [77]. Members of the genus *Calanus* are amongst the largest copepods and can comprise as much as 90% of the dry weight of mezozooplankton in regions of the North and Celtic Seas [77].

The calanoid copepod species *Calanus finmarchicus* is one of the most important large zooplankton species in the subarctic waters of the North Atlantic [78] and dominates the dry weight of the mesozooplankton in the northern regions. *C*. *finmarchicus* does not reside year-round in the North Sea (although it did so in the 1960s [57]), and its population is replenished each spring by advection of late-stage individuals mainly originating from an overwintering stock located beyond the shelf edge, in the deep Atlantic and Norwegian Sea, particularly the Faroe-Shetland Channel [79, 80], which makes this species presence in this area very susceptible to circulation changes.

Over the past four decades there has been a pronounced decline in its abundance in the North Sea: in 1962, the species represented 80% of the total *Calanus*, and it used to dominate the spring time biomass, whereas it represented only 20% of the genus by the beginning of the 2000s [8, 11, 12, 81, 82].

**Fig 1:**
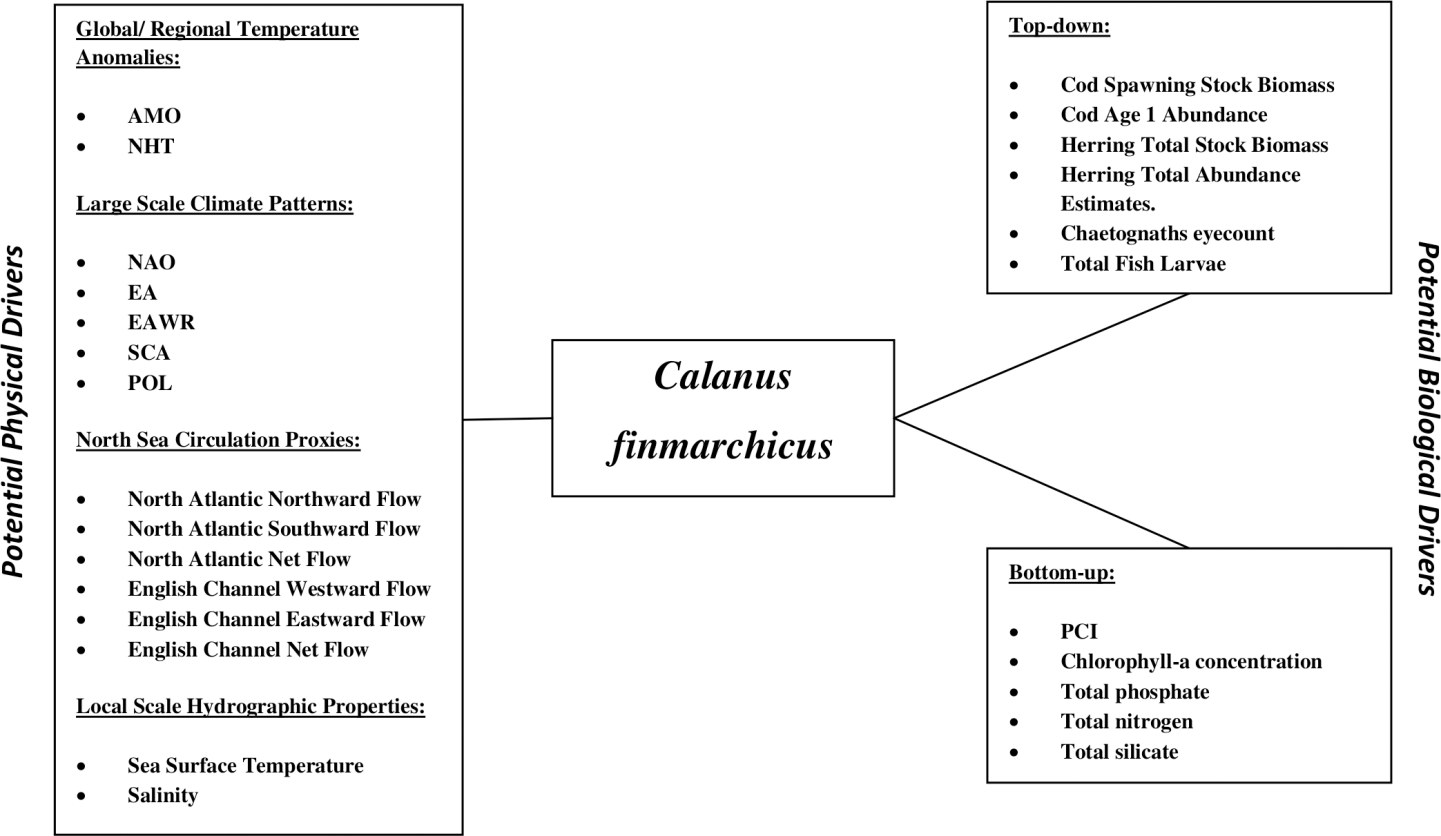
Variables identified as potential drivers of the abundance of *C*. *finmarchicus*. Potential drivers have been sectioned into physical, on the left, and biological, on the right. Biological variables have subsequently been divided into two further groups, top-down and bottom-up, which are positioned above and below *C*. *finmarchicus* respectively. The data used in this research article are listed in Table 1.

*Calanus finmarchicus* is subjected to a range of drivers, both physical and trophic (top-down and bottom-up) (Fig 1) [81]. From eggs to adults, it is a primary source of food for commercially important fish species, such as cod (larvae), herring, lesser sandeel, mackerel, blue whiting, anchovy and chaetognaths, and as such its dynamics have been extensively studied [28, 63, 83–89]. *C*. *finmarchicus* feeds on a range of microplankton including diatoms, dinoflagellates, ciliates, coccolithophores and rotifers [90, 91]. Sea surface temperature determines the geographical, vertical, seasonal distributions, the development, and physiology of calanoid copepods, and can be particularly relevant for *C*. *finmarchicus*, as this species is at the edge of its thermal niche in the North Sea [11, 23, 57, 58, 71, 73, 77, 92–95]. Because this species reproduces in the Norwegian Sea and individuals are transported from there into the North Sea, marine circulation and the atmospheric patterns driving it are crucial for its geographical distribution [11, 22, 79, 80, 96].

While the literature cited above indicates that this species can potentially be influenced by a variety of drivers, many of the studies mentioned use only part of the possible drivers, which might result in a partial picture [97]. Whilst most research traditionally focuses on a single driver (e.g. NAO, SST, or fishery impacts), in this work we use the GP-based methodology on a large (86) collection of variables, encompassing both physical and trophic, bottom-up and top-down, potential drivers of *C*. *finmarchicus* (Table 1), without any *a priori* assumptions on the population’s drivers.

**Table 1:** Timeseries used in this study. The links to the data sets used are shown in S3 File.

Dataset		Units	Period	Gaps	Frequency	Area	Source
**Physical variables**	North Atlantic Oscillation (NAO)		1950–2012	No	Monthly	North Eastern Atlantic	**NOAA-CPC**
	East Atlantic Pattern (EA)		1950–2012	No	Monthly	North Eastern Atlantic	**NOAA-CPC**
	East Atlantic West Russia Pattern (EAWR)		1950–2012	No	Monthly	North Eastern Atlantic	**NOAA-CPC**
	Scandinavian Pattern (SCA)		1950–2012	No	Monthly	North Eastern Atlantic	**NOAA-CPC**
	Polar Eurasia Pattern (POL)		1950–2012	No	Monthly	North Eastern Atlantic	**NOAA-CPC**
	Atlantic Multidecadal Oscillation (AMO)		1948–2012	No	Monthly	North Atlantic	**NOAA-PSD**
	Northern Hemisphere Temperature (NHT)		1850–2012	No	Monthly	Northern Hemisphere	**NOAA-anomalies**
	N. Atlantic Southward Flow	Sverdrup	1970–2012	No	Monthly	Orkney-Norway	**NORWegian ECOlogical Model System (NORWECOM)**
	N. Atlantic Northward Flow	Sverdrup	1970–2012	No	Monthly	Orkney-Norway	**NORWegian ECOlogical Model System (NORWECOM)**
	N. Atlantic Net Flow	Sverdrup	1970–2012	No	Monthly	Orkney-Norway	**NORWegian ECOlogical Model System (NORWECOM)**
	English Channel Eastward Flow	Sverdrup	1970–2012	No	Monthly	Dover Strait	**NORWegian ECOlogical Model System (NORWECOM)**
	English Channel Westward Flow	Sverdrup	1970–2012	No	Monthly	Dover Strait	**NORWegian ECOlogical Model System (NORWECOM)**
	English Channel Net Flow	Sverdrup	1970–2012	No	Monthly	Dover Strait	**NORWegian ECOlogical Model System (NORWECOM)**
	Sea Surface Temperature (SST)	°C	1891–2012	Yes	Monthly	55 to 60°N and -2.5 to 9°E	**ICES (surface data)**
	Sea Surface Salinity (SSS)	PSU	1891–2012	Yes	Monthly	55 to 60°N and -2.5 to 9°E	**ICES (surface data)**
**Biological variables**							
**Bottom-up drivers**	Total Nitrogen (N)	μmol/l	1970–2012	Yes	Monthly	55 to 60°N and -2.5 to 9°E	**ICES (Bottle data)**
	Total Phosphorus (P)	μmol/l	1969–2012	Yes	Monthly	55 to 60°N and -2.5 to 9°E	**ICES (Bottle data)**
	Silicate (SiO_4_)	μmol/l	1958–2012	Yes	Monthly	55 to 60°N and -2.5 to 9°E	**ICES (Bottle data)**
	Chlorophyll-a (Chl-a)	μg/l	1961–2012	Yes	Monthly	55 to 60°N and -2.5 to 9°E	**ICES (Bottle data)**
	Phytoplankton Colour Index (PCI)	1–5 scale	1958–2011	Yes	Monthly	CPR areas C1 and C255 to 58°N and -3 to 11°E	**Sir Alister Hardy Foundation for Ocean Science**
**Top-down drivers**	Chaetognaths Eyecount	Mean	1958–2011	Yes	Monthly	CPR areas C1 and C255 to 58°N and -3 to 11°E	**Sir Alister Hardy Foundation for Ocean Science**
	Total Fish Larvae	abundance	1958–2011	Yes	Monthly	CPR areas C1 and C255 to 58°N and -3 to 11°E	**Sir Alister Hardy Foundation for Ocean Science**
	Herring *(Claupea harengus)* Total Stock Abundance	estimate	1947–2011	No	Annual	Subarea IV	**ICES HAWG**
	Herring *(Claupea harengus)*Total Stock Biomass	tonnes	1947–2011	No	Annual	Subarea IV	**ICES HAWG**
	Cod *(Gadus morhua)* Aged 1 year	‘000’s	1963–2011	No	Annual	Subarea IV and Divisions IIIa and VIId	**ICES WGNSSK**
	Cod *(Gadus morhua)* Spawning Stock Biomass	tonnes	1963–2011	No	Annual	Subarea IV and Divisions IIIa and VIId	**ICES WGNSSK**
	**Target Variable:**						
	*Calanus finmarchicus*	Mean	1958–2011	Yes	Monthly	CPR areas C1 and C255 to 58°N and -3 to 11°E	**Sir Alister Hardy Foundation for Ocean Science**

## Materials and Methods

### Overall analysis approach

**Fig 2:**
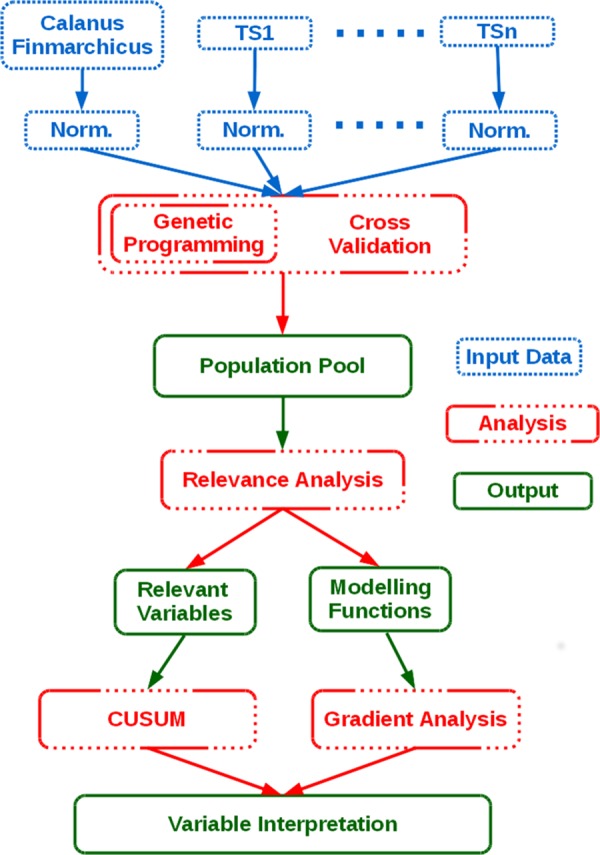
The analysis approach used in this work. The figure summarizes the proposed analysis approach, where TS1, …, TSn are the time series shown in Table 1, Norm is the data normalisation step needed to analyse time series with different magnitudes, the Genetic Programming and the Cross-Validation respectively generate and validate the functions that approximate the target variable, the Relevant Analysis identifies the relevant ecological variables and the modelling functions capable to express the target variable, and finally the Gradient Analysis identifies the role of the relevant variables in relation to the target variable. CuSUM is the cumulative sums analysis used to identify the starting year of the regime shift in the target and relevant variables.

The statistical approach used in this work is based on Genetic Programming, complemented by additional statistical analyses (Fig 2). The Genetic Programming approach is a data driven methodology, which has no *a priori* assumptions on the relationship between the data—these relationships are selected through an evolutionary process lasting hundreds of generations. This evolutionary/selection process is validated through a cross validation procedure. The Relevance Analysis complements the selection of the variables by identifying those that occur with a frequency superior to chance, which are therefore deemed relevant for the approximation of the target variable. The Gradient Analysis is specific to the identification of the drivers of the target variable since it provides essential information for causal associations, i.e., the direction (direct or inverse) between target and relevant variables. The CuSUM analysis identifies the year of a regime shifts, and is specific to the North Sea case study, as this sea was involved in an ecological regime shift in the 1980s.

The details of each analysis are given in the next sections. The mathematical in depth information is provided in S1 File.

### Genetic programming

Genetic Programming (GP) is an evolutionary computation approach that generates solutions starting from an initial population of randomly generated functions, based on a set of variables, mathematical primitives, and constants [44–46]. As the name suggests, the initial solutions are improved by miming the selection processes that occur naturally in biological systems. This is done through an iterative process encompassing multiple generations, during which the Selection, Crossover, Clonation, and/or Mutation GP operators are applied.

This process is explained in detail in S1 File. The initial population consisted of 1000 individuals (i.e., functions) and the evolutionary process lasted for at most 500 generations, when the stop criterion was met. To evaluate the fitness of the evolved individuals we used the Root Mean Squared Error (RMSE) [98] between the target and the evolved individuals.

Similarly to the approach described in [99], the GP based procedure was coupled with a Cross-Validation framework (CV) [98, 100, 101], with the aim of selecting a set of approximating functions that generalise the target variable’s dynamics.

The CV process randomly splits each time series into two disjoint subsets: (i) the training subset used to run the GP procedure in order to evolve the functions which approximate the target variable; (ii) the validation subset used to evaluate the approximation capability of the evolved functions. Since the time indices of the validation subset are not involved in the training phase (training and validation subsets being disjoint), the generalization capability of an evolved function is defined as the error obtained by predicting the values of the target variable on the time indices of the validation subset. The smaller the prediction error, the more the function generalises and is therefore more suitable for explaining the dynamics of the target variable. The best generalising functions are selected to become part of a group of functions approximating the target variable, named *population-pool*. The heterogeneous mathematical forms of the population-pools individuals are normal in the context of GP, as the diversity of the evolved genetic material (genotype) captures the complexity of the target variable [102]. In fact, functions with different genotypes (function syntax) can produce similar phenotype effects (function semantic), as genotype diversity, together with phenotype convergence, indicates the existence of multiple solutions for the same problem [102].

All the population-pool functions were subsequently analysed for identifying the variables *relevant* to the target time series.

### Relevance Analysis

A variable is deemed ***relevant*** if it appears in the individuals of the population-pool more times than by chance. On the contrary, not relevant variables should be considered randomly selected by the GP procedure and for this reason they are not suitable for explaining the behaviour of the target variable.

The problem of identifying whether a variable is relevant or not can be formalised as a Bernoulli trial [103] defined on the number of functions of the population-pool. In the proposed experiments, the assumption that all the variables have the same probability to appear among the individuals of the population-pool is rejected with p-value equal to 0.001 according to Johnson [104]. According to the proposed Bernoulli trial, this p-value corresponds to a variable occurrence greater than or equal to a threshold variable occurrence. This means that in our study all the variables whose occurrence among the individuals of the population-pool is greater than or equal to the threshold are deemed relevant (for details see S1 File).

Some approximating functions, or individuals, in the population-pool contain only relevant variables; other functions contain both relevant and not relevant variables. The former individuals should be considered a reliable set of functions for modelling the target variable, since they do not contain random components; for this reason, in the remainder of this article the individuals that contain only relevant variables are named ***modelling functions*** (listed in S1 File). In contrast, the individuals containing random components (not relevant variables) are deemed not valid for generalising the dynamics of the target variable [52].

The relevant variables and the modelling functions extracted from the population-pool through the k-fold cross-validation framework and the proposed statistical analysis reduce the possibility that random information contained in the evolved functions produces over-fitting of the investigated time-series.

### Gradient Analysis

The GP procedure, as all multivariate or correlation analyses, can identify the relationships but cannot provide information on the corresponding causal direction, i.e., it does not have the ability to distinguish the internal dynamics in trophic chains, in particular drivers from “drivees”. This distinction needs to be made through the analysis of the relationships between approximating functions, original time series, and target variable [105].

This is a common problem in the analysis of populations within food webs. To solve this we have added to the results of the GP procedure a gradient analysis with the purpose to identify the role of each relevant variable with respect to the target variable dynamics. In this work, the partial derivatives of all the modelling functions have been analysed based on the finite difference approach in order to estimate the function gradient, and from this, to infer whether a relevant variable is directly or inversely proportional to the target variable.

### Cumulative Sums Analysis

The relevant variables were tested for a change point in their mean to further compare their significance to the target variable. Cumulative Sums analysis (CuSUM) was used to determine the year of the most prominent shift in the time series. This method consists of plotting the cumulative sum of standardised values over time. Each value of the series is subtracted from the mean of the time series, resulting in a new time series of residuals which are used for the calculation of the cumulative sum. The interpretation is based on the slope of the line on the chart: a constant deviation from the mean of the time series shows a constant slope. Persistent changes from the mean of the times series cause a persistent change of the slope [66, 106].

### Case Study: *Calanus finmarchicus* in the North Sea

#### Data

A total of 26 environmental variables representing large and local scale, trophic or physical potential drivers of *C*. *finmarchicus* were used in this study (Table 1). Climatic and hydrographic variables were further divided into seasonal averages because we wanted to evaluate the effect different seasons can have on zooplankton abundance [95]. Therefore, a total of 86 time series were used in this experiment. The multiplication of time series over several seasons is not considered as a duplication of information, as the GP based approach simply identifies the variables that are relevant in relation to *C*. *finmarchicus* and ignores all other variables, independently from the number of variables used.

Plankton data were obtained from the Sir Alister Hardy Foundation for Ocean Science (SAHFOS) database for the Continuous Plankton Recorder (CPR), survey regions C1 and C2, located in the central North Sea. The CPR survey method is described in detail in [107–110]. The CPR plankton data (abundances) were separated into the target variable (*Calanus finmarchicus*), bottom-up (phytoplankton colour index (PCI)—proxy for food availability), and top-down (chaetognaths and total fish larvae—proxies for predation) trophic variables.

Herring *(Clupea harengus)* and cod *(Gadus morhua)* estimates were chosen because they are major predators of *C*. *finmarchicus* [63, 84, 111]. Yearly fisheries stock assessment data for herring total stock biomass (TSB), herring abundance (sum of all age groups), cod spawning stock biomass (SSB), and 1 year old cod (cod1) were retrieved from the annual stock-assessment reports for the North Sea, ICES area IV [112, 113], which corresponds to the area selected for *C*. *finmarchicus*. Cod spawning stock biomass values were used as a proxy for the amount of larvae (which feed on *C*. *finmarchicus*) which could be produced by the adult cod that year, with a higher SSB inferring more cod larvae production. Cod stock assessments do not estimate year 0 age groups, therefore estimates of 1-year-old cod were also used as a proxy for the abundance of larvae present the year before. These two variables were used to represent the predation pressure of larval cod, as that is the key life stage which feeds on plankton.

Oceanographic data were downloaded from the International Council for the Exploration of the Sea (ICES) Oceanographic Database for the same area as CPR survey regions C1 and C2. Chemical and biological variables, total nitrogen, total phosphorus, silicate (SiO_4_), all as μmol/l, and chlorophyll-a (μg/l) were downloaded from the CTD and Bottle Data database (Table 1). Total nitrogen, phosphorus and silicate are considered proxies for primary productivity.

Seawater temperature and hydrography affect *C*. *finmarchicus* biogeography [39, 64]. SST (°C) and surface salinity (PSU) were downloaded from the ICES Surface Data database, which consists of CTD, Bottle and Underway/Pump data collected at depths < 10m (Table 1).

Additional temperature measurements included the Northern Hemisphere Temperature (NHT) index, which is a large scale temperature index, defined as the combination of land and sea surface temperature anomalies over the entire northern hemisphere and the Atlantic Multidecadal Oscillation (AMO), a regional index that describes long-term variations in sea surface temperature of the Atlantic Ocean [114] (Table 1).

Large-scale climatic patterns influence circulation in the North Atlantic and North Sea and therefore need to be considered as potential drivers of *C*. *finmarchicus* abundance. For this work, the following climate patterns where chosen: North Atlantic Oscillation (NAO), East Atlantic Pattern (EA), East Atlantic/ West Russia Pattern (EAWR), Scandinavian Pattern (SCA), The Polar/ Eurasian pattern (POL). These data were downloaded from http://www.cpc.ncep.noaa.gov/data/teledoc/telecontents.shtml (Table 1).

North Sea circulation and flow data are from the NORWegian ECOlogical Model system (NORWECOM). NORWECOM is a coupled 3D physical, chemical and biological model, validated for the North Sea and the Skagerrak [115]. Average monthly transports through an east-west section from Utsira (Norway) to the Orkney Islands along 59°17’N in the northern North Sea (N. Atlantic inflow) and a longitudinal section through the English Channel in the Dover Straits along 0°E (English Channel inflow) were computed, and values for inflow, outflow and net flow were used. Inflow represents southwards flow through the Utsira to Orkney transect and eastward through the Dover Strait, whilst outflow represents northward and westward flow respectively.

The data used shows some bias which should be mentioned. It has been suggested that the CPR shallow sampling depth may not be adequate at sampling species absolute numbers [116, 117], and those which migrate beneath the thermocline, such as *C*. *finmarchicus* [118]. However, analysis has shown the CPR is a consistent semi quantitative index of phyto- and zoo-plankton abundance in comparison to satellite data [119] and to other planktonic sampling techniques [107, 109, 120, 121], and in particular that it provides an accurate representation of both spatial and temporal patterns of *C*. *finmarchicus* [122].

Fishery stock assessment data are based on model estimates from all available data for that species and are reviewed extensively before publishing; they are favoured over total landing values because they take into account fishing effort [123]. The yearly-averaged nutrient data may not contain 12 monthly values, meaning some months may be underrepresented, especially at the start of the series. Cod SSB and cod1 are not actually feeding on *C*. *finmarchicus* larvae (see Discussion), and they were used as proxies for larval production. These two variables were chosen because they were the best larval cod proxy data that we could find.

#### Data manipulation

Due to missing data and different begin-end dates in the time series, this study used the period in common, 39 years from 1972 to 2011. Yearly averages which consisted of 3 or less monthly values were excluded from the GP-based model to improve accuracy. The removal of these years is the cause of the gaps in the approximating functions output.

The 39 time indices were split into k = 13 folds corresponding to 3 years each according to the k-fold Cross-Validation design, and the GP procedure was run 50 times per fold, each time randomly sampling 85% of the time indices.

ICES chemical and biological data recorded below the depth of 20m were excluded from analysis, due to the towed depth of the CPR and the productive surface layer above the thermocline generally forming at 15–20 m in the summer months in the North Sea [68]. Fisheries data were available at an annual frequency whilst all other variables were downloaded at or averaged to a monthly frequency. Because of this, all monthly times series were yearly or seasonally averaged prior to analysis. Seasonally averaged data means that the averages were calculated over the 3 months of each season, as follows. Climate seasons for NAO, EA, EA.WR, SCA, POL, AMO and NHT, were defined as winter (December-February), spring (March-May), summer (June-August) and autumn (September-November), whilst seasons for hydrographic variables (Atlantic flow, English channel flow, SST and salinity) lag one month behind climatic, i.e. winter is January-March, etc. [124]. Nutrient and biological data were averaged annually with no seasons implied.

The time series presented in Table 1 refer to the observed data with their own units and scales. All the time series were normalised by dividing each value by the maximum value of that time series [125]. The time series having only positive values were normalised in the range [0,1], while the time series having both positive and negative values were normalised in the range [–1,1]. Without this normalisation approach, time series whose values are in the order of 10^7^ (e.g., cod and herring abundance) could not be compared to time series whose values were in the order of 10^1^ (e.g., hydrographic parameters, climate indices). This normalisation approach discards the scale of the time series, and allows focusing only on the time series dynamics. In this way, the proposed analysis approach can select the relevant variables capable to describe the dynamics of the target variable.

## Results

### Application of the GP-based analysis to *Calanus finmarchicus* abundance in the North Sea

#### 1. Identification of the relevant variables

In this work, we have used GP to identify the variables that are relevant for approximating the abundance of *Calanus finmarchicus* in the North Sea, using 86 biological and physical variables, chosen because they are potential drivers of *C*. *finmarchicus*. The GP parameters and the Cross validation design used in the case study are detailed in S1 File.

At the end of all the GP/cross-validation iterations, the population-pool consisted of 104 functions. The average validation error of these functions, resulting from the cross-validation process, was 0.102 with a standard deviation equal to 0.082.

**Fig 3:**
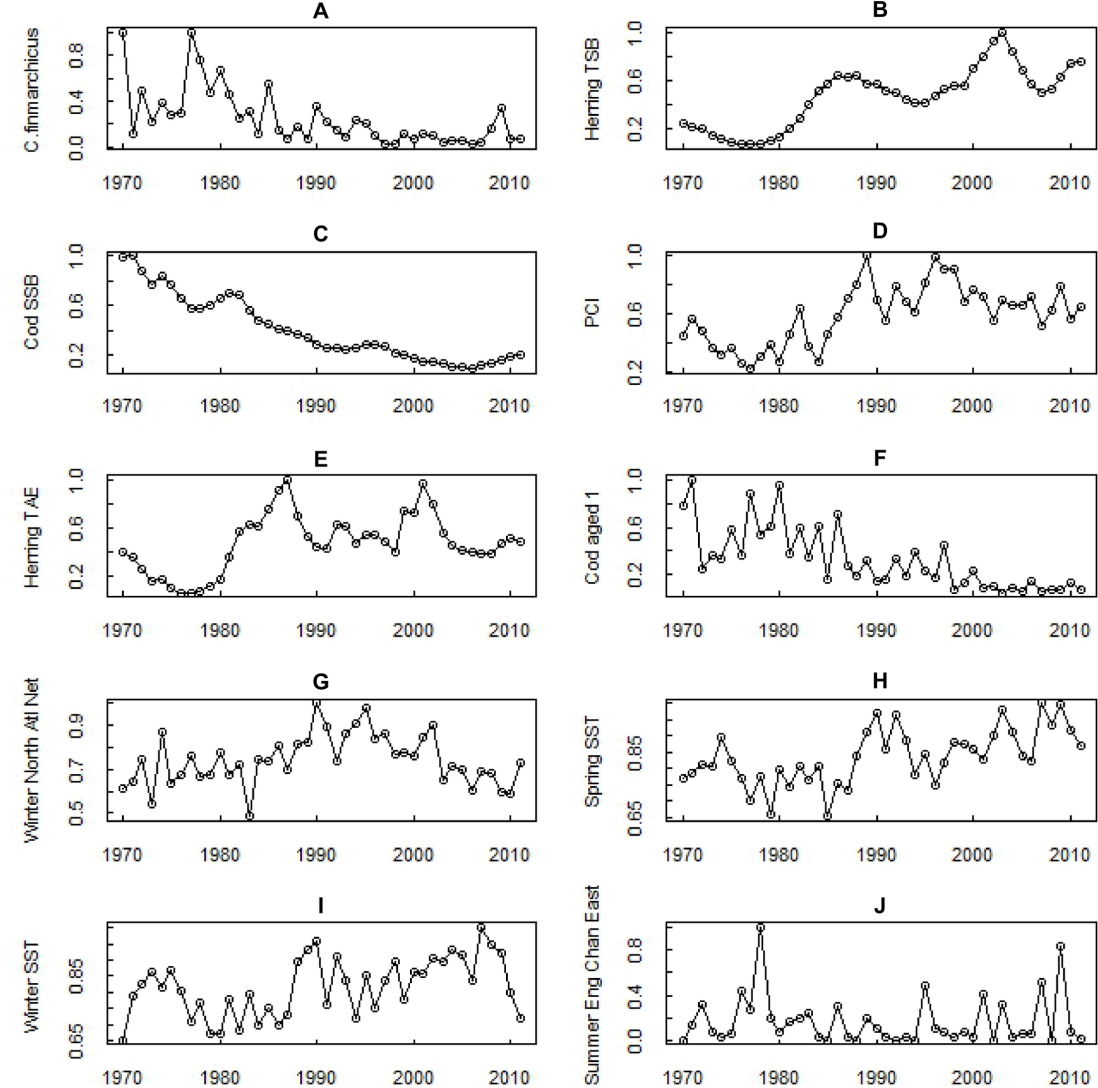
Time series of *Calanus finmarchicus* and the 9 relevant variables identified by the GP procedure. The time series are ordered from left to right of most frequently occurring. All time series were normalised by dividing each value by the time series maximum value before the GP process.

The relevance analysis of the population pool identified the variables that occurred with a frequency superior to chance among the approximating functions of the population-pool. According to the statistic test based on the Bernoulli trial (see S1 File for details), the variables whose occurrence frequency was larger than 7 were deemed relevant. Out of the original 86 potential drivers, 9 variables, statistically relevant for the approximation of *C*. *finmarchicus*, where thus identified. These 9 relevant variables (6, once seasons and multiple datasets are combined, e.g., herring biomass and abundance represent 1 potential driver, herring) are, in order of frequency of occurrence in the final population-pool: **herring** (total stock biomass and total abundance estimates), **cod** (spawning stock biomass and abundance at age 1), **phytoplankton** (as represented by the PCI), **SST** (spring and winter), and two circulation variables, **N. Atlantic net flow** in winter (wNAtlNET) and **English Channel eastward flow** in summer (smEnglChanE) (Table 2, Fig 3). It is worth noticing that a) the relevant variables encompass both top-down/ bottom-up (predators, prey proxies) biological and physical (climate, temperature, circulation,) potential drivers, and b) they do not encompass large-scale climate indices.

**Table 2:** Relevant variables selected by the Genetic Programming based methodology combined with the relevance analysis, the abbreviations used in this article, and the frequency of occurrence of each variable in the 104 approximating functions in the population pool. The last column indicates the type of relationship between the relevant variables and *C*. *finmarchicus*, identified with the gradient analysis.

Variable:	Short name	Occurrence	Direction of relationship
Herring Total Stock Biomass	HerringTSB	40	inverse
Cod Spawning Stock Biomass	CodSSB	38	*variable*
Phytoplankton Colour Index	PCI	35	inverse
Herring Total Abundance	HerringTAE	15	inverse
Cod Aged 1	Cod1	13	*direct*
Winter North Atlantic Net Flow	wNAtlNET	9	*direct*
Spring SST	spSST	8	inverse
Winter SST	wSST	7	inverse
Summer English Channel Eastwards Flow	smEnglChanE	7	inverse

#### 2. Temporal shifts in the relevant variables

**Fig 4:**
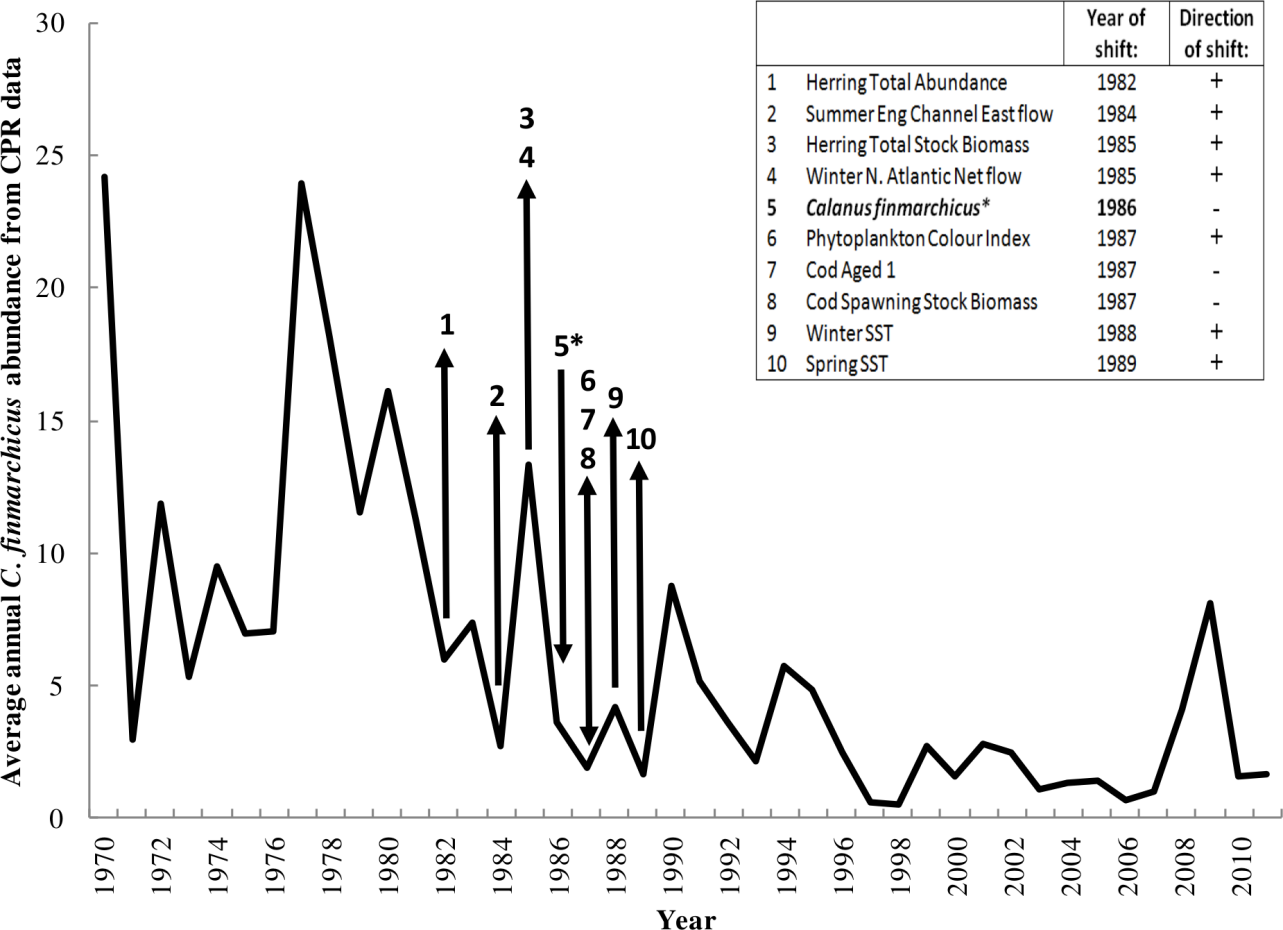
Sequences of abrupt shifts in the North Sea. Time series of *C*. *finmarchicus* average annual abundance, with arrows indicating the years of the regime shifts, detected using cumulative sums analysis both on this species and in the 9 variables identified as relevant by GP. The table in the insert specifies the year of the shift and shows its direction: + meaning an increase,—meaning a decrease.

In order to further evaluate the selection of the 9 variables by the GP procedure, each variable was tested for an abrupt shift in the mean using Cumulative Sums analysis. All 9 time series showed a shift during the 1980s (Fig 4) (see S2 File for each variables CuSUM plot).

A shift towards a lower mean abundance of *C*. *finmarchicus* was detected in 1986. This happened after shifts towards higher means in herring (1982/85), summer English Channel eastward flow (1984) and winter N. Atlantic net flow (1985). After the downward shift in *C*. *finmarchicus*, PCI saw an increase in 1987, while cod (both age 1 and spawning stock biomass) concurrently decreased. Winter and spring SST shifted upwards in 1988 and 1989 respectively.

#### 3. Gradient analysis of the relevant variables

In order to understand the nature of the relationship of the 9 relevant variables with *C*. *finmarchicus*, gradient analysis was used. This analysis provides information on the type of relationship (direct, inverse, variable) between each relevant variable and *C*. *finmarchicus*, which gives important information for identifying the likely drivers.

The results of this analysis are presented in the last column of Table 2. All relevant variables are inversely related to *C*. *finmarchicus*, with the exception of N. Atlantic net flow (positively related), and cod, which is either positively related (cod1) or variable (SSB).

This analysis indicated that some of the relevant variables are very unlikely drivers of *C*. *finmarchicus*.

PCI showed an indirect relationship with *C*. *finmarchicus*. In the studies period PCI (a prey proxy) increased, yet *C*. *finmarchicus* decreased. Further, the CuSUM analysis showed a (positive) shift in PCI after a (negative) shift in *C*. *finmarchicus*. Both analyses suggest that PCI is not driving, but, if anything, is driven by *C*. *finmarchicus*, but see the Discussion. Hence, PCI has been eliminated as a likely driver.

Cod age 1 showed a direct relationship with *C*. *finmarchicus*. This predator (proxy) decreased in the studied period, yet *C*. *finmarchicus* decreased as well, indicating that it was not driven by it (but see the Discussion for more information). Hence Cod age 1 was eliminated as a likely driver.

Cod SSB had a variable relationship showing no consistent relationship with *C*. *finmarchicus*, hence it was also eliminated as a likely driver, but see the Discussion for more details on this variable.

The **summer English Channel eastward flow** was eliminated as a potential driver over magnitude considerations. The overall flow exchange with the N. Atlantic through the Strait of Dover (English Channel flow) is about 1/10^th^ of the flow exchange through the Faroe-Shetland transect (N. Atlantic flow) [126]. However, due to the normalisation process applied in this work, the differences in the variables magnitude are lost, and all variables belong to the same range. While this variable resulted relevant, its small magnitude indicates that it is not a likely driver.

**Fig 5:**
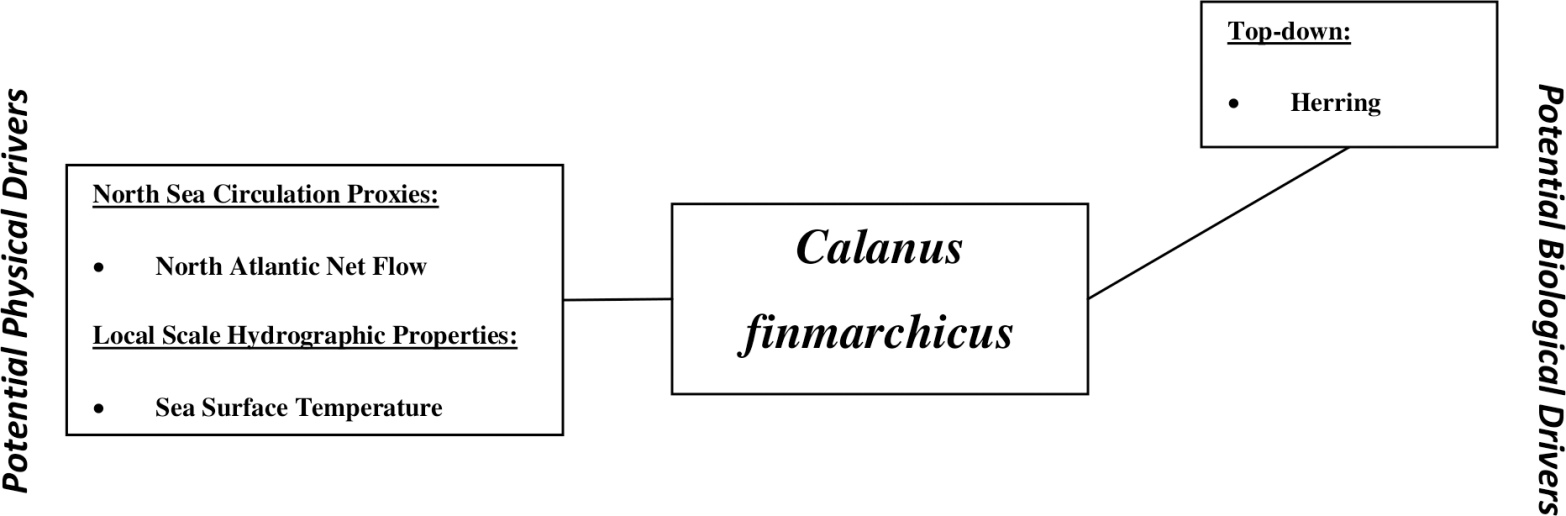
The new conceptual model of potential drivers of *Calanus finmarchicus* abundance deduced from the GP method supplemented by the relevance and gradient analyses. The new model is composed of two physical variables, North Atlantic net flow and Sea Surface Temperature, and one biological variable, Herring, which is recognised as a top-down driver.

All the above variables remain nevertheless relevant and can be used to approximate *C*. *finmarchicus* abundance and for forecasting experiments,

With the use of gradient analysis, supported by a literature review, 5 (3 once combined) of the 9 relevant variables selected by the relevance analysis can be identified as likely drivers: **herring** (total abundance estimate and total stock biomass), **SST** (winter and spring) and **N. Atlantic net flow** in winter, shown in Fig 5.

## Discussion

### 1. Evaluation of the GP-based methodology

The aim of the proposed analysis methodology is the identification of the relevant information needed to explain the dynamics of the investigated ecosystem. This is obtained by evolving a set of mathematical functions capable to generalise the behaviour of the target variable (i.e. *C*. *finmarchicus* abundance in the case study).

In the design of the statistical methodology used in this work we took special care in producing generalized (*vs* overfitting) results, in significantly selecting the relevant variables, and in correctly identifying potential drivers.

The generalising capability of the evolved functions was obtained by coupling the Genetic Programming procedure with a k-fold Cross-Validation (CV) design, which provided a prediction error for each evolved function. The individuals with the smallest prediction error (i.e., the greatest generalising capability) were selected for the final population pool.

The relevance analysis provided the tool to statistically identify within the population pool relevant (not occurring by chance) variables for the target species. Additional experiments were ran, which used randomly generated time series together with the ecological variables. During these experiments, the artificial variables were never selected as relevant, thus confirming the reliability of the proposed statistical analysis.

While the GP/cross validation plus relevance analysis methodology can identify significant relevant variables (in the case of *C*. *finmarchicus*, nine out of the 86 initially selected possible drivers), a present limitation of this methodology is that it cannot provide information on the role they play for explaining the dynamics of the target variable. This is a limitation common to all multivariate or correlation analyses. In the *C*. *finmarchicus* application, we approached the question of the causal relationship among relevant and target variables with the gradient analysis, by analysing the partial derivatives of the modelling functions, i.e., the members of the population-pool involving only relevant variables. The Gradient Analysis provided information on the relationship between the target variable and the relevant variables incorporated in the modelling functions.

The results achieved by applying this methodology to *Calanus finmarchicus* in the North Sea are coherent with what can be seen in the literature (see next section), which validates this approach.

### 2. Application to *Calanus finmarchicus* in the N. Sea

In this section, we combine the results of GP/CV, relevant, gradient and cumulative sums analyses with a literature review, and discuss their relationship with C. *finmarchicus* to explain its decline over a 40 year period. The set of variables selected by the GP analysis is justified and evaluated in order to demonstrate the potential use of this method in identifying drivers within a marine system.

#### Relevant variables

The 9 variables shown in Table 2 and Fig 3 encompass both physical (temperature and circulation) and biological variables (both top-down (predators) and bottom-up (prey) proxies). All the relevant variables are associated to modelling functions (listed in S1 File), which can be used to approximate or forecast the target variable’s abundance.

#### Relevant physical variables

Out of the large selection of potential physical drivers (Fig 1 and Table 1), only SST and circulation proxies (the flow in/out of the North Sea) were identified as relevant. It is quite interesting that none of the large-scale climate patterns (e.g., NAO, NHT…) were identified by the GP model as relevant variables. These findings contradict a large body of research cited earlier suggesting that large-scale climate anomalies are the main drivers of marine ecosystems. However, our results may have been the outcome of having both large scale and local drivers as model inputs. In other words, when there is availability of both data types, local drivers seem to be more important factors to population dynamics, even if they are controlled by larger scale factors.

**Winter and spring SST** (Fig 3H & 3I) increased steadily over the time series, and the gradient analysis identified an inverse relationship with *C*. *finmarchicus* (Table 2), meaning that a temperature increase corresponded to a decrease in this species. It has been suggested that the increased SST in the North Sea has affected the physiology and development rates of *C*. *finmarchicus* individuals [58], since this species, whose thermal critical boundary is around 10° C, is at the edge of its niche in the North Sea [81, 127]. The temperature increase is proposed to be the main cause of a northwards range shift of this and other species, and of its decrease in the study area by several authors [2, 5, 8, 14, 19, 81, 128, 129], although other studies have found *C*. *finmarchicus* is still abundant below the thermocline [118], indeed that under higher SST the relative abundance of *C*. *finmarchicus* in deeper water increases [57].This suggests that the North Sea population might have moved to a cooler, deeper zone as SST increased, reducing its abundance in the CPR time-series. While increasing spring SST may affect the development and growth of *C*. *finmarchicus* [58, 130, 131], changes in winter SST may also be associated to circulation changes, which also can affect plankton abundance in the sea [15, 132]. SST in fact acts as an overarching driver, not only directly affecting *C*. *finmarchicus*, but also its predators, its prey [105, 133, 134] and the surrounding habitat. For example, an increase in winter SST is suspected to affect the overwintering habitat of *C*. *finmarchicus* in areas such as the Norwegian Sea deepwater. An increase in SST and deeper ocean temperatures reduces the available area for *C*. *finmarchicus* to overwinter causing a decrease in the North Sea population by affecting reproductive capabilities and leading to less individuals being advected into the North Sea in spring [79, 96].

**N. Atlantic *net* flow** is a circulation proxy for the net amount of flow leaving-entering the North Sea via a transect running from Scotland to Norway. The **winter** N. Atlantic net flow was northward (positive) throughout the time series, as the flow leaving was always greater than that entering the N. Sea. The flow intensity varied over time, increasing until 1990, and then declining (Fig 3G). The *C*. *finmarchicus* time series shows an opposite trend, steadily declining since the late 1970s, then staying low before a year of higher abundance at the end (Fig 3), and in fact the relationship between these variables is overall inverse (Table 2). This inverse relationship between *C*. *finmarchicus* and net flow can be related to the transport of zooplankton into/out of the North Sea. The increasing overall flow to the north in the first half of the series could in fact have significantly contributed to reducing the number of *C*. *finmarchicus* that were re-entering the North Sea [11, 12, 22]. An increase in winter northward flow could also transport warmer water further northwards which could contribute to the northward shift of this cold-water species and its reduction in the North Sea as well as impacting the size of cold water overwintering areas such as the Norwegian Sea deepwater [8, 96]. It has also been suggested that transport of warmer water into the North Sea has led to increased competition for food from warm water species such *Calanus helgolandicus* [77].

The **English Channel flow** represents the flow entering/exiting the southern North Sea via the Strait of Dover (eastward = entering). The summer English Channel eastward flow (Fig 3J) has been eliminated as a likely driver due to the evaluation of its magnitude and potential influence on the North Sea (see Results). It remains, however, a relevant variable for approximating *C*. *finmarchicus* abundance.

#### Relevant biological variables: bottom–up

The bottom-up drivers included chlorophyll, PCI, and nutrients. Of these, only PCI appeared as relevant, but an unlikely driver of *C*. *finmarchicus*.

The **Phytoplankton colour index (PCI)** provides an index based on the colour that the accumulation of green chlorophyll pigments gives to the CPR filtering silk. It is considered to be a semi-quantitative measurement of phytoplankton abundance [120], which is a main food source for *C*. *finmarchicus* [90, 91]. PCI saw an abrupt increase during the 1980s (Fig 3D) despite concurrent decreasing nutrient concentrations [135], possibly because of changes in the phytoplankton predator community over the past 40 years [7]. With an increase in phytoplankton one would expect an increase in the abundance of their predator *C*. *finmarchicus*. However, our gradient analysis shows the opposite, i.e. a direct relationship (Table 2). This confirms the findings by several studies in the North Sea on increased phytoplankton abundance while *C*. *finmarchicus* decreased [19, 70, 136–138]. This discordance suggests that while the feeding environment in the North Sea may have changed to one seemingly more favourable for *C*. *finmarchicus*, changes in other drivers might be more important for this particular species. Alternatively, it can be considered that PCI might not reflect *C*. *finmarchicus* diet, being an aggregate index which provides no information on species (for example, the abundance of several diatom species decreased in this period [139]). Finally, PCI might have increased because of reduced pressure by *C*. *finmarchicus*. Both our analyses and the literature review indicate that it is unlikely that PCI is a direct driver of *C*. *finmarchicus* (if anything, the direct relationship with *C*. *finmarchicus* suggests that PCI is driven by it), for which reason it was discarded as a driver. However, because of its possible links with overarching physical drivers, such as SST, whose increase could boost phytoplankton biomass but adversely affect *C*. *finmarchicus* abundance [7, 136], PCI remains very relevant for approximating and possibly predicting *C*. *finmarchicus* abundance, as indicated by its frequency in the GP output.

#### Relevant biological variables: top-down

Only herring and cod where selected as relevant by the GP approach, while other predators, such as chaetognaths or fish larvae were not selected at all. These two species were the most frequently selected variables, which suggest they are very relevant for *C*. *finmarchicus* abundance; however, the overall patterns are very different, and only herring can be considered as a driver.

**Herrings** are planktivorous throughout its life, and *C*. *finmarchicus* can be an important prey [84, 140]. **Herring biomass (TSB)** is the most frequently selected variable by the GP program, occurring 40 times, and also the selection of **herring abundance (TAE**) is very high, occurring 15 times (Table 2). The gradient analysis shows that both herring parameters are inversely related to *C*. *finmarchicus* (Table 2), which is consistent with the hypothesis that herring can drive *C*. *finmarchicus* abundance through predation. This inverse relationship is likely related to the large herring population increase over the time series whilst *C*. *finmarchicus* declined at a similar rate (Fig 3B & 3E). The persistent selection of herring by the GP model suggests that herring predation pressure could be one of the most important drivers of *C*. *finmarchicus*.

**Cod spawning stock biomass (SSB) and cod age 1 abundance (cod1)** were also selected as relevant by the algorithm with high frequencies (38 and 13 times respectively, Table 2). The cod variables in fact showed a similar rate of decline as *C*. *finmarchicus* over the time series (Fig 3C & 3F). The cause of the decline in cod and cod larvae has been attributed to overfishing and the miss timing of larvae cod and their food, as *C*. *finmarchicus* declined and was replaced by other warmer water species of zooplankton which have different life cycles and nutrition quality [36, 82, 133, 134], and to more complex dynamics involving competition with herring, internal feedbacks in the ecosystems, and the reversals of predator-prey roles [43, 74, 141]. Moreover, in this study we used cod SSB and cod age-1 as proxies for cod larvae, for which there were no data available. This is however a gross simplification; in fact, while *C*. *finmarchicus* is a major prey for cod larvae, juvenile and adult cods prey on a wide variety of epifaunal species or species associated with hard substrates, and also on the planktivorous herring and so do not directly drive *C*. *finmarchicus* [133, 142]. The gradient analysis harmonises with these studies by showing a direct relationship between this copepod and cod aged 1, and a positive but variable (not constant through the series) relationship with cod biomass (Table 2). Both the analyses and the literature review propose that cod is not driving *C*. *finmarchicus*. If anything, it is driven by it. Cod SSB and cod-1 still remain very relevant variables for approximating *C*. *finmarchicus* abundance, and can possibly be used for predicting it.

Overall, the literature review supports and gives an ecological explanation to the selection of the environmental variables relevant for *C*. *finmarchicus* abundance

#### Temporal changes in the North Sea (the 1980s regime shift)

Abrupt shifts in the means of the relevant variables were analysed with the CuSUM analysis, in order to determine the temporal sequence of changes in the identified variables and thus propose an explaination of *C*. *finmarchicus*’s decline (Fig 4). All variables presented a shift in the 1980s. This is a well-known period in time and a large number of studies have addressed the ecological regime shift that involved the North Sea during this period [67, 70] as well as other European basins [15, 68, 69, 124].

It has been proposed that the overfishing of cod caused a reversal in the trophic roles between herring and cod [43, 74]. Herring was released from predation pressure when cod numbers declined and the herring population was then able to increase, shifting around **1982–5** (Fig 4). Herring predation on larval cod subsequently increased, stopping the trophic roles from switching back, even if there was reduced fishing pressure on cod [74]. The increased herring population also predated on zooplankton at a greater magnitude than cod, as herring are planktivorous throughout their lives [63, 84]. This was then intensified by a positive shift in the N. Atlantic net (overall northward) flow in **1985** (Fig 4) which might have contributed to reduced advection of *C*. *finmarchicus* from the Norwegian Sea into the North Sea, as well as an increase in central North Sea SST by transporting more heat northwards, possibly impacting the formation of overwintering habitats. The synergistic impacts of these drivers might have caused the abundance of *C*. *finmarchicus* to shift to a lower state around **1986** (Fig 4), before an increase (**1987**) in phytoplankton (PCI), possibly released from the copepod predation or responding positively to increased SST, and a decrease in cod biomass and age 1 larvae (**1987**), possibly related to their prey reduction and overfishing, occurred (Fig 4). The shift in *C*. *finmarchicus* preceded the increase in winter and spring SST in **1988/89**, both of which might have contributed to the continuing decline in the abundance of *C*. *finmarchicus* in the subsequent years by affecting their geographical range, overwintering habitat space, physiology, food availability and competition with other zooplankton species.

In this proposed scenario, the decline in *C*.*finmarchicus* may have been initiated by overfishing and a change in top-down drivers, whose impact has then been magnified by a change in the Atlantic net flow in the North Sea (physical drivers). As the population of *C*. *finmarchicus* declined, other drivers, such as the increasing SST, may have caused its abundance to decrease further. This switch in drivers might explain why, while herring have experienced recruitment failures since 2000 [37], the *C*. *finmarchicus* population still declined further. The importance of synergistic and time-delayed roles in driving regime shifts is examined in Conversi *et al* [2], and this work suggests that such a scenario could be identified more frequently than usually thought, provided that the statistical analyses include diverse types of drivers.

## Conclusions

This study has shown how the Genetic Programming based methodology can select a small number of variables from a large initial pool. In the application on *C*. *finmarchicus* in the North Sea, the ecological meaning of the relevant variables (SST, North Atlantic net flux, English Channel flux, PCI, cod and herring) is backed up by a review of the literature. The analysis of the temporal shifts in these variables can explain *C*. *finmarchicus* decline within the 41 years study period.

Moreover, the capability of the proposed analysis approach to produce multiple mathematical models, which use a few variables to approximate the same target, can also be very useful for future robust forecasting applications. These applications could include how the target population respond to climate change using predicted conditions, for example in a global warming scenario. This application could be very important for ecosystem studies and management and we are currently investigating its feasibility. Additional studies of potential interest include comparing this data-driven methodology with knowledge-based mechanistic models.

In the *C*. *finmarchicus* application, the list of relevant variables selected by the GP-based analysis, fine-tuned with the relevance and gradient analyses provided 3 likely drivers of this species abundance (Herring, Natl flow, SST) out of the 86 initially selected. This result highlights the importance of both physical and biological drivers on the abundance of this species in the North Sea, and increases our understanding of how climate, circulation, and predation all play a part. The ability to identify relevant drivers indicates future applications for using this GP-based analysis within marine ecosystems, and shows that it can shed light on ecosystem events such as regime shifts.

The application of GP to marine ecology is very novel. With this work we propose that this method holds promises in the near future for the highly non-linear field of ecology, in the same way as the consolidated GP-based methodologies discussed in the introduction have obtained relevant results in many scientific fields, like for example robotics, physics, stock market analysis, and medicine.

## Supporting Information

S1 File Detailed information on the Genetic Programming parameters used in this study. (PDF)

S2 File Example of an approximating function produced by the Genetic Programming based approach, and cumulative sums analysis results. (PDF)

S3 File Data availability information. (DOCX)

## References

[1] Link JS, Yemane D, Shannon LJ, Coll M, Shin YJ, Hill L, et al Relating marine ecosystem indicators to fishing and environmental drivers: an elucidation of contrasting responses. Ices Journal of Marine Science. 2010;67(4):787–95. 10.1093/icesjms/fsp258 .

[2] Conversi A, Dakos V, Gardmark A, Ling S, Folke C, Mumby PJ, et al A holistic view of marine regime shifts. Philosophical Transactions of the Royal Society B-Biological Sciences. 2015;370(1659):8 10.1098/rstb.2013.0279 .

[3] Nelson GC, Bennett E, Berhe AA, Cassman K, DeFries R, Dietz T, et al Anthropogenic drivers of ecosystem change: An overview. Ecology and Society. 2006;11(2). .

[4] Pershing A, Mills K, Record N, Stamieszkin K, Wurtzell K, Byron C, et al Evaluating trophic cascades as drivers of regime shifts in different ocean ecosystems. Philosphical Transactions of the Royal Society B 2015;370(20130265). 10.1098/rstb.2013.0272

[5] Kirby RR, Beaugrand G. Trophic amplification of climate warming. Proceedings of the Royal Society B-Biological Sciences. 2009;276(1676):4095–103. 10.1098/rspb.2009.1320 . PMC2821349 19740882

[6] Lauria V, Attrill MJ, Pinnegar JK, Brown A, Edwards M, Votier SC. Influence of Climate Change and Trophic Coupling across Four Trophic Levels in the Celtic Sea. Plos One. 2012;7(10). 10.1371/journal.pone.0047408 . PMC3472987 23091621

[7] Beaugrand G, Reid PC. Long-term changes in phytoplankton, zooplankton and salmon related to climate. Global Change Biology. 2003;9(6):801–17. 10.1046/j.1365-2486.2003.00632.x .

[8] Beaugrand G, Edwards M, Brander K, Luczak C, Ibanez F. Causes and projections of abrupt climate-driven ecosystem shifts in the North Atlantic. Ecology Letters. 2008;11(11):1157–68. 10.1111/j.1461-0248.2008.01218.x . 18647332

[9] Kirby RR, Beaugrand G, Lindley JA, Richardson AJ, Edwards M, Reid PC. Climate effects and benthic-pelagic coupling in the North Sea. Mar Ecol-Prog Ser. 2007;330:31–8. 10.3354/meps330031 .

[10] Planque B, Taylor AH. Long-term changes in zooplankton and the climate of the North Atlantic. Ices Journal of Marine Science. 1998;55(4):644–54. 10.1006/jmsc.1998.0390 .

[11] Greene CH, Pershing AJ, Conversi A, Planque B, Hannah C, Sameoto D, et al Trans-Atlantic responses of Calanus finmarchicus populations to basin-scale forcing associated with the North Atlantic Oscillation. Progress in Oceanography. 2003;58(2–4):301–12. 10.1016/j.pocean.2003.08.009 .

[12] Reid PC, Edwards M, Beaugrand G, Skogen M, Stevens D. Periodic changes in the zooplankton of the North Sea during the twentieth century linked to oceanic inflow. Fisheries Oceanography. 2003;12(4‐5):260–9.

[13] Frederiksen M, Edwards M, Richardson AJ, Halliday NC, Wanless S. From plankton to top predators: bottom-up control of a marine food web across four trophic levels. Journal of Animal Ecology. 2006;75(6):1259–68. 10.1111/j.1365-2656.2006.01148.x . 17032358

[14] Edwards M, Beaugrand G, Helaouët P, Alheit J, Coombs SH. Marine ecosystem response to the Atlantic Multidecadal Oscillation. Plos One. 2013;8:e57212 10.1371/journal.pone.0057212 23460832 PMC3584106

[15] Beaugrand G, Conversi A, Chiba S, Edwards M, Fonda-Umani S, Greene C, et al Synchronous marine pelagic regime shifts in the Northern Hemisphere. Philosphical Transactions of the Royal Society B 2015;370(1659). 10.1098/rstb.2013.0272

[16] Mackas D, Greve W, Edwards M, Chiba S, Tadokoro K, Eloire D, et al Changing zooplankton seasonality in a changing ocean: Comparing time series of zooplankton phenology. Progress in Oceanography. 2012;97:31–62. 10.1016/j.pocean.2011.11.005 .

[17] Conversi A, Hameed S. Common signals between physical and atmospheric variables and zooplankton biomass in the Subarctic Pacific. ICES Journal of Marine Science: Journal du Conseil. 1998;55(4):739–47.

[18] Lauria V, Attrill MJ, Brown A, Edwards M, Votier SC. Regional variation in the impact of climate change: evidence that bottom-up regulation from plankton to seabirds is weak in parts of the Northeast Atlantic. Marine Ecology Progress Series. 2013;488:11–22.

[19] Harris V, Edwards M, Olhede SC. Multidecadal Atlantic climate variability and its impact on marine pelagic communities. Journal of Marine Systems. 2014;133:55–69. 10.1016/j.jmarsys.2013.07.001 .

[20] Drinkwater KF, Beaugrand G, Kaeriyama M, Kim S, Ottersen G, Perry RI, et al On the processes linking climate to ecosystem changes. Journal of Marine Systems. 2010;79(3–4):374–88. 10.1016/j.jmarsys.2008.12.014.

[21] Pitois SG, Lynam CP, Jansen T, Halliday N, Edwards M. Bottom-up effects of climate on fish populations: data from the Continuous Plankton Recorder. Marine Ecology Progress Series. 2012;456:169–86. 10.3354/meps09710 .

[22] Hatun H, Payne MR, Beaugrand G, Reid PC, Sando AB, Drange H, et al Large bio-geographical shifts in the north-eastern Altantic Ocean: from the subpolar gyre, via plankton, to blue whiting and pilot whales. Progress in Oceanography. 2009;80:149–62.

[23] Beaugrand G. Theoretical basis for predicting climate-induced abrupt shifts in the oceans. Philosophical Transactions of the Royal Society of London B: Biological Sciences. 2015;370(1659):20130264.

[24] Casini M, Hjelm J, Molinero JC, Lovgren J, Cardinale M, Bartolino V, et al Trophic cascades promote threshold-like shifts in pelagic marine ecosystems. Proc Natl Acad Sci U S A. 2009;106(1):197–202. 10.1073/pnas.0806649105 . 19109431 PMC2629246

[25] Frank KT, Petrie B, Shackell NL, Choi JS. Reconciling differences in trophic control in mid-latitude marine ecosystems. Ecology Letters. 2006;9(10):1096–105. 10.1111/j.1461-0248.2006.00961.x . 16972873

[26] Frank KT, Petrie B, Shackell NL. The ups and downs of trophic control in continental shelf ecosystems. Trends in Ecology & Evolution. 2007;22(5):236–42. 10.1016/j.tree.2007.03.002 . 17350714

[27] Lassalle G, Lobry J, Le Loc'h F, Mackinson S, Sanchez F, Tomczak MT, et al Ecosystem status and functioning: searching for rules of thumb using an intersite comparison of food-web models of Northeast Atlantic continental shelves. Ices Journal of Marine Science. 2013;70(1):135–49. 10.1093/icesjms/fss168 .

[28] Fauchald P, Skov H, Skern-Mauritzen M, Johns D, Tveraa T. Wasp-Waist Interactions in the North Sea Ecosystem. Plos One. 2011;6(7). 10.1371/journal.pone.0022729 . PMC3145753 21829494

[29] Frank KT, Petrie B, Choi JS, Leggett WC. Trophic cascades in a formerly cod-dominated ecosystem. Science. 2005;308:1621–3. 15947186 10.1126/science.1113075

[30] Pauly D, Christensen V, Dalsgaard J, Froese R, Torres F. Fishing Down Marine Food Webs. Science. 1998;279(5352):860–3. 10.1126/science.279.5352.860 9452385

[31] Rosenblatt AE, Heithaus MR, Mather ME, Matich P, Nifong JC, Ripple WJ, et al. The roles of large top predators in coastal ecosystems: New insights from Long Term Ecological Research. 2013.

[32] de Young B, Barange M, Beaugrand G, Harris R, Perry RI, Scheffer M, et al Regime shifts in marine ecosystems: detection, prediction and management. Trends in Ecology & Evolution. 2008;23(7):402–9. 10.1016/j.tree.2008.03.008 . 18501990

[33] Möllmann C, Muller-Karulis B, Kornilovs G, St John MA. Effects of climate and overfishing on zooplankton dynamics and ecosystem structure: regime shifts, trophic cascade, and feedback coops in a simple ecosystem. Ices Journal of Marine Science. 2008;65(3):302–10. 10.1093/icesjms/fsm197 .

[34] Mackinson S, Daskalov G, Heymans JJ, Neira S, Arancibia H, Zetina-Rejon M, et al Which forcing factors fit? Using ecosystem models to investigate the relative influence of fishing and changes in primary productivity on the dynamics of marine ecosystems. Ecological Modelling. 2009;220(21):2972–87. 10.1016/j.ecolmodel.2008.10.021 .

[35] Casini M, Bartolino V, Molinero JC, Kornilovs G. Linking fisheries, trophic interactions and climate: threshold dynamics drive herring Clupea harengus growth in the central Baltic Sea. Marine Ecology Progress Series. 2010;413:241–52. 10.3354/meps08592 .

[36] Olsen EM, Ottersen G, Llope M, Chan K-S, Beaugrand G, Stenseth NC. Spawning stock and recruitment in North Sea cod shaped by food and climate. Proceedings of the Royal Society B: Biological Sciences. 2011;278(1705):504–10. 10.1098/rspb.2010.1465 20810442 PMC3025682

[37] Payne MR, Hatfield EMC, Dickey-Collas M, Falkenhaug T, Gallego A, Gröger J, et al Recruitment in a changing environment: the 2000s North Sea herring recruitment failure. Ices Journal of Marine Science. 2009;66(2):272–7. 10.1093/icesjms/fsn211

[38] Mullin M, Conversi A. Biomasses of euphausiids and smaller zooplankton in the California Current—geographic and interannual comparisons relative to the Pacific whiting, Merluccius productus, fishery. Fishery Bulletin. 1989;87(3):633–44. .

[39] Greene CH, Meyer-Gutbrod E, Monger BC, McGarry LP, Pershing AJ, Belkin IM, et al Remote climate forcing of decadal-scale regime shifts in Northwest Atlantic shelf ecosystems. Limnol Oceanogr. 2013;58(3):803–16.

[40] Greene CH, Monger BC, McGarry LP, Connelly MD, Schnepf NR, Pershing AJ, et al Recent Arctic Climate Change and Its Remote Forcing of Northwest Atlantic Shelf Ecosystems. Oceanography. 2012;25(3):208–13. .

[41] Kirby RR, Beaugrand G, Lindley JA. Synergistic effects of climate and fishing in a marine ecosystem. Ecosystems. 2009;12:548–61.

[42] Hunt GL Jr, McKinnell S. Interplay between top-down, bottom-up, and wasp-waist control in marine ecosystems. Progress in Oceanography. 2006;68(2):115–24.

[43] Hjermann DO, Fisher JAD, Rouyer T, Frank KT, Stenseth NC. Spatial analysis of North Sea cod recruitment: concurrent effects of changes in spawning stock biomass, temperature and herring abundance. Marine Ecology Progress Series. 2013;480:263–+. 10.3354/meps10315 .

[44] Koza JR. Genetic Programming: On the Programming of Computers by Means of Natural Selection Cambridge: MIT Press; 1992. p.

[45] Poli R, Langdon WB, McPhee NF. A Feild Guide to Genetic Programming. Available: http://www.gp-field-guide.org.uk/. 2008.

[46] Koza JR. Human-competitive results produced by genetic programming. Genetic Programming and Evolvable Machines. 2010;11(3–4):251–84.

[47] Parker G, Gulcu B, Ieee. EVOLVING PREDATOR CONTROL PROGRAMS FOR A HEXAPOD ROBOT PURSUING A PREY. New York: Ieee; 2008. 411–7 p.

[48] Kala R. Multi-robot path planning using co-evolutionary genetic programming. Expert Systems with Applications. 2012;39(3):3817–31. 10.1016/j.eswa.2011.09.090 .

[49] Schmidt M, Lipson H. Distilling Free-Form Natural Laws from Experimental Data. Science. 2009;324(5923):81–5. 10.1126/science.1165893 19342586

[50] Huang CJ, Chen PW, Pan WT. Using multi-stage data mining technique to build forecast model for Taiwan stocks. Neural Computing & Applications. 2012;21(8):2057–63. 10.1007/s00521-011-0628-0 .

[51] Lones MA, Smith SL, Alty JE, Lacy SE, Possin KL, Jamieson DRS, et al Evolving Classifiers to Recognize the Movement Characteristics of Parkinson's Disease Patients. Evolutionary Computation, IEEE Transactions on. 2014;18(4):559–76. 10.1109/TEVC.2013.2281532

[52] Marini S, Conversi A. Understanding Zooplankton Long Term Variability through Genetic Programming In: Giacobini M, Vanneschi L, Bush W, editors. Evolutionary Computation, Machine Learning and Data Mining in Bioinformatics. Lecture Notes in Computer Science. 7246: Springer Berlin Heidelberg; 2012 p. 50–61.

[53] Muttil N, Lee JHW. Genetic programming for analysis and real-time prediction of coastal algal blooms. Ecological Modelling. 2005;189(3–4):363–76. 10.1016/j.ecolmodel.2005.03.018 .

[54] Hongqing C, Recknagel F, Orr PT. Parameter Optimization Algorithms for Evolving Rule Models Applied to Freshwater Ecosystems. Evolutionary Computation, IEEE Transactions on. 2014;18(6):793–806. 10.1109/TEVC.2013.2286404

[55] Vladislavleva E, Friedrich T, Neumann F, Wagner M. Predicting the energy output of wind farms based on weather data: Important variables and their correlation. Renewable Energy. 2013;50:236–43. 10.1016/j.renene.2012.06.036.

[56] Record NR, Pershing AJ, Runge JA, Mayo CA, Monger BC, Chen C. Improving ecological forecasts of copepod community dynamics using genetic algorithms. Journal of Marine Systems. 2010;82(3):96–110. 10.1016/j.jmarsys.2010.04.001 .

[57] Maar M, Møller EF, Gürkan Z, Jónasdóttir SH, Nielsen TG. Sensitivity of Calanus spp. copepods to environmental changes in the North Sea using life-stage structured models. Progress in Oceanography. 2013;111:24–37. 10.1016/j.pocean.2012.10.004.

[58] Møller EF, Maar M, Jónasdóttir SH, Nielsen TG, Tönnesson K. The effect of changes in temperature and food on the development of Calanus finmarchicus and Calanus helgolandicus populations. Limnology and Oceanography. 2012;57(1):211–20. 10.4319/lo.2012.57.1.0211

[59] Augusto DA, Barbosa HJ, editors. Symbolic regression via genetic programming. Neural Networks, 2000 Proceedings Sixth Brazilian Symposium on; 2000: IEEE.

[60] Vladislavleva E, Smits G, Den Hertog D. On the importance of data balancing for symbolic regression. Evolutionary Computation, IEEE Transactions on Evolutionary Computation. 2010;14(2):252–77.

[61] OSPAR. Quality status report 2010. OSPAR Commission. 2010.

[62] OSPAR. Quality status report 2000. OSPAR Commission. 2000.

[63] Raab K, Nagelkerke LAJ, Boeree C, Rijnsdorp AD, Temming A, Dickey-Collas M. Dietary overlap between the potential competitors herring, sprat and anchovy in the North Sea. Marine Ecology Progress Series. 2012;470:101–11. 10.3354/meps09919 .

[64] Beaugrand G, Luczak C, Edwards M. Rapid biogeographical plankton shifts in the North Atlantic Ocean. Global Change Biology. 2009;15(7):1790–803. 10.1111/j.1365-2486.2009.01848.x .

[65] Biggs R, Blenckner T, Folke C, Gordon L, Norström A, Nyström M, et al Regime shifts In: Hastings A, Gross L, editors. Encyclopedia of theoretical ecology University of California Press, Berkeley 2012 p. 609–17.

[66] Andersen T, Carstensen J, Hernandez-Garcia E, Duarte CM. Ecological thresholds and regime shifts: approaches to identification. Trends in Ecology & Evolution. 2009;24(1):49–57. 18952317 10.1016/j.tree.2008.07.014

[67] Beaugrand G. The North Sea regime shift: evidence, causes, mechanisms and consequences. Progress in Oceanography. 2004;60(2–4):245–62. 10.1016/j.pocean.2004.02.018 .

[68] Dippner JW, Möller C, Hänninen J. Regime shifts in North Sea and Baltic Sea: a comparison. Journal of Marine Systems. 2012;105–108:115–22.

[69] Alheit J, Mollmann C, Dutz J, Kornilovs G, Loewe P, Mohrholz V, et al Synchronous ecological regime shifts in the central Baltic and the North Sea in the late 1980s. Ices Journal of Marine Science. 2005;62(7):1205–15. 10.1016/j.icejms.2005.04.024 .

[70] Alvarez-Fernandez S, Lindeboom H, Meesters E. Temporal changes in plankton of the North Sea: community shifts and environmental drivers. Marine Ecology Progress Series. 2012;462:21–38. 10.3354/meps09817 .

[71] Beaugrand G, Reid PC. Relationships between North Atlantic salmon, plankton, and hydroclimatic change in the Northeast Atlantic. ICES Journal of Marine Science. 2012;69:1549–62.

[72] Luczak C, Beaugrand G, Lindley JA, Dewarumez JM, Dubois PJ, Kirby RR. North Sea ecosystem change from swimming crabs to seagulls. Biology Letters. 2012;8(5):821–4. 10.1098/rsbl.2012.0474 . 22764111 PMC3441004

[73] Beaugrand G, Ibanez F. Monitoring marine plankton ecosystems (2): long-term changes in North Sea calanoid copepods in relation to hydro-meteorological variability. Marine Ecology Progress Series. 2004;284:35–47.

[74] Fauchald P. Predator-prey reversal: A possible mechanism for ecosystem hysteresis in the North Sea? Ecology. 2010;91(8):2191–7. 10.1890/09-1500.1 . 20836439

[75] Beaugrand G, Harlay X, Edwards M. Detecting plankton shifts in the North Sea: a new abrupt ecosystem shift between 1996 and 2003. Marine Ecology Progress Series. 2014;502:85–104. 10.3354/meps10693 .

[76] Alheit J. Consequences of regime shifts for marine food webs. Int J Earth Sci. 2009;98(2):261–8. 10.1007/s00531-007-0232-9 .

[77] Helaouet P, Beaugrand G. Macroecology of Calanus finmarchicus and C-helgolandicus in the North Atlantic Ocean and adjacent seas. Mar Ecol-Prog Ser. 2007;345:147–65. 10.3354/meps06775 .

[78] Melle W, Runge J, Head E, Plourde S, Castellani C, Licandro P, et al The North Atlantic Ocean as habitat for Calanus finmarchicus: Environmental factors and life history traits. Progress in Oceanography. 2014;129:244–84. 10.1016/j.pocean.2014.04.026 .

[79] Heath MR, Backhaus JO, Richardson K, McKenzie E, Slagstad D, Beare D, et al Climate fluctuations and the spring invasion of the North Sea by Calanus finmarchicus. Fisheries Oceanography. 1999;8(s1):163–76.

[80] Harms I, Heath M, Bryant A, Backhaus J, Hainbucher D. Modelling the Northeast Atlantic circulation: implications for the spring invasion of shelf regions by Calanus finmarchicus. ICES Journal of Marine Science: Journal du Conseil. 2000;57(6):1694–707.

[81] Helaouët P, Beaugrand G, Reid PC. Macrophysiology of Calanus finmarchicus in the North Atlantic Ocean. Progress in Oceanography. 2011;91(3):217–28. 10.1016/j.pocean.2010.11.003.

[82] Beaugrand G, Brander KM, Lindley JA, Souissi S, Reid PC. Plankton effect on cod recruitment in the North Sea. Nature. 2003;426(6967):661–4. 10.1038/nature02164 . 14668864

[83] Tande KS, Miller CB. Population dynamics of Calanus in the North Atlantic: results from the trans-Atlantic study of Calanus finmarchicus. Ices Journal of Marine Science. 2000;57(6):1527–. 10.1006/jmsc.2000.0983 .

[84] Prokopchuk I, Sentyabov E. Diets of herring, mackerel, and blue whiting in the Norwegian Sea in relation to Calanus finmarchicus distribution and temperature conditions. Ices Journal of Marine Science. 2006;63(1):117–27. 10.1016/j.icesjms.2005.08.005 .

[85] Simonsen CS, Munk P, Folkvord A, Pedersen SA. Feeding ecology of Greenland halibut and sandeel larvae off West Greenland. Marine Biology. 2006;149(4):937–52. 10.1007/s00227-005-0172-5 .

[86] van Deurs M, van Hal R, Tomczak MT, Jonasdottir SH, Dolmer P. Recruitment of lesser sandeel Ammodytes marinus in relation to density dependence and zooplankton composition. Marine Ecology Progress Series. 2009;381:249–58. 10.3354/meps07960 .

[87] Raab K, Nagelkerke LAJ, Boeree C, Rijnsdorp AD, Temming A, Dickey-Collas M. Anchovy Engraulis encrasicolus diet in the North and Baltic Seas. Journal of Sea Research. 2011;65(1):131–40. 10.1016/j.seares.2010.09.002 .

[88] Kehayias G, Michaloudi E, Koutrakis E. Feeding and predation impact of chaetognaths in the north Aegean Sea (Strymonikos and Ierissos Gulfs). Journal of the Marine Biological Association of the United Kingdom. 2005;85(6):1525–32. 10.1017/s0025315405012737 .

[89] Petursdottir H, Falk-Petersen S, Gislason A. Trophic interactions of meso- and macrozooplankton and fish in the Iceland Sea as evaluated by fatty acid and stable isotope analysis. Ices Journal of Marine Science. 2012;69(7):1277–88. 10.1093/icesjms/fss125 .

[90] Nejstgaard JC, Gismervik I, Solberg PT. Feeding and reproduction by Calanus finmarchicus, and microzooplankton grazing during mesocosm blooms of diatoms and the coccolithophore Emiliania huxleyi. Marine Ecology Progress Series. 1997;147(1–3):197–217. 10.3354/meps147197 .

[91] Castellani C, Irigoien X, Mayor DJ, Harris RP, Wilson D. Feeding of Calanus finmarchicus and Oithona similis on the microplankton assemblage in the Irminger Sea, North Atlantic. Journal of Plankton Research. 2008;30(10):1095–116. 10.1093/plankt/fbn074 .

[92] Beaugrand G, Goberville E, Luczak C, Kirby RR. Marine biological shifts and climate. Proceedings of the Royal Society B: Biological Sciences. 2014;281(1783):20133350 10.1098/rspb.2013.3350 24718760 PMC3996605

[93] Planque B, Batten SD. Calanus finmarchicus in the North Atlantic: the year of Calanus in the context of interdecadal change. Ices Journal of Marine Science. 2000;57(6):1528–35. 10.1006/jmsc.2000.0970 .

[94] Reygondeau G, Beaugrand G. Water column stability and Calanus finmarchicus. Journal of Plankton Research. 2011;33(1):119–36. 10.1093/plankt/fbq091 .

[95] Usov N, Kutcheva I, Primakov I, Martynova D. Every species is good in its season: Do the shifts in the annual temperature dynamics affect the phenology of the zooplankton species in the White Sea? Hydrobiologia. 2013;706(1):11–33. 10.1007/s10750-012-1435-z .

[96] Kimmel DG, Hameed S. Update on the relationship between the North Atlantic Oscillation and Calanus finmarchicus. Marine Ecology Progress Series. 2008;366:111–7. 10.3354/meps07523 .

[97] O'Connor MI, Holding JM, Kappel CV, Duarte CM, Brander K, Brown CJ, et al Strengthening confidence in climate change impact science. Global Ecology and Biogeography. 2015;24(1):64–76. 10.1111/geb.12218 .

[98] Bergmeir C, Benítez JM. On the use of cross-validation for time series predictor evaluation. Information Sciences. 2012;191:192–213. 10.1016/j.ins.2011.12.028.

[99] Corgnati L, Mazzei L, Marini S, Aliani S, Conversi A, Griffa A, et al., editors. Automated Gelatinous Zooplankton Acquisition and Recognition. Computer Vision for Analysis of Underwater Imagery (CVAUI), ICPR 2014 2014 24–24 Aug. 2014.

[100] Efron B, Gong G. A Leisurely Look at the Bootstrap, the Jackknife, and Cross-Validation. The American Statistician. 1983;37(1):36–48. 10.1080/00031305.1983.10483087

[101] Kohavi R, editor A study of cross-validation and bootstrap for accuracy estimation and model selection. Proceedings of the 14th International joint conference on Artificial intelligence; 1995; Montreal, Quebec, Canada: Morgan Kaufmann Publishers Inc.

[102] Corriveau G, Guilbault R, Tahan A, Sabourin R. Review of phenotypic diversity formulations for diagnostic tool. Applied Soft Computing. 2013;13(1):9–26. 10.1016/j.asoc.2012.08.046.

[103] Lefebvre M. Applied probability and statistics: Springer Science & Business Media; 2007.

[104] Johnson VE. Revised standards for statistical evidence. Proceedings of the National Academy of Sciences. 2013;110(48):19313–7. 10.1073/pnas.1313476110 PMC3845140 24218581

[105] Moloney CL, St John MA, Denman KL, Karl DM, Koster FW, Sundby S, et al Weaving marine food webs from end to end under global change. Journal of Marine Systems. 2011;84(3–4):106–16. 10.1016/j.jmarsys.2010.06.012 .

[106] Ibanez F, Fromentin J, Castel J. Application of the cumulated function to the processing of chronological data in oceanography. Comptes Rendus De L Academie Des Sciences Serie Iii-Sciences De La Vie-Life Sciences. 1993;316(8):745–8.

[107] Richardson AJ, Walne AW, John AWG, Jonas TD, Lindley JA, Sims DW, et al Using continuous plankton recorder data. Progress in Oceanography. 2006;68(1):27–74. 10.1016/j.pocean.2005.09.011.

[108] Batten SD, Clark R, Flinkman J, Hays GC, John E, John AWG, et al CPR sampling: the technical background, materials and methods, consistency and comparability. Progress in Oceanography. 2003;58(2–4):193–215. 10.1016/j.poccan.2003.08.004 .

[109] Clark RA, Frid CLJ, Batten S. A critical comparison of two long-term zooplankton time series from the central-west North Sea. Journal of Plankton Research. 2001;23(1):27–39. 10.1093/plankt/23.1.27 .

[110] Colebrook J. The continuous plankton recorder survey: automatic data processing methods. Bulletin Marine Ecology. 1975;8:123–42.

[111] Heath MR, Lough RG. A synthesis of large-scale patterns in the planktonic prey of larval and juvenile cod (Gadus morhua). Fisheries Oceanography. 2007;16(2):169–85. 10.1111/j.1365-2419.2006.00423.x .

[112] ICES. Report of the Herring Assessment Working Group for the Area South of 62 N (HAWG), 13–22 March 2012. Copenhagen, Denmark. ICES CM 2012/ACOM:06.835 pp.: 2012.

[113] ICES. Report of the Working Group on the Assessment of Demersal Stocks in the North Sea and Skagerrak (WGNSSK), 27 April—3 May 2012. 2012.

[114] Knight JR, Folland CK, Scaife AA. Climate impacts of the Atlantic Multidecadal Oscillation. Geophysical Research Letters. 2006;33(17). 10.1029/2006gl026242 .

[115] Søiland H, Skogen MD. Validation of a three-dimensional biophysical model using nutrient observations in the North Sea. ICES Journal of Marine Science: Journal du Conseil. 2000;57(4):816–23. 10.1006/jmsc.2000.0567

[116] Owens NJP, Hosie GW, Batten SD, Edwards M, Johns DG, Beaugrand G. All plankton sampling systems underestimate abundance: Response to "Continuous plankton recorder underestimates zooplankton abundance" by JW Dippner and M. Krause. Journal of Marine Systems. 2013;128:240–2. 10.1016/j.jmarsys.2013.05.003 .

[117] Dippner JW, Krause M. Continuous plankton recorder underestimates zooplankton abundance. Journal of Marine Systems. 2013;111:263–8. 10.1016/j.jmarsys.2012.09.009 .

[118] Jonasdottir SH, Koski M. Biological processes in the North Sea: comparison of Calanus helgolandicus and Calanus finmarchicus vertical distribution and production. Journal of Plankton Research. 2011;33(1):85–103. 10.1093/plankt/fbq085 .

[119] Raitsos DE, Reid PC, Lavender SJ, Edwards M, Richardson AJ. Extending the SeaWiFS chlorophyll data set back 50 years in the northeast Atlantic. Geophysical Research Letters. 2005;32(6):L06603 10.1029/2005GL022484

[120] Batten SD, Walne AW, Edwards M, Groom SB. Phytoplankton biomass from continuous plankton recorder data: an assessment of the phytoplankton colour index. Journal of Plankton Research. 2003;25(7):697–702. 10.1093/plankt/25.7.697 .

[121] Richardson AJ, John EH, Irigoien X, Harris RP, Hays GC. How well does the Continuous Plankton Recorder (CPR) sample zooplankton? A comparison with the Longhurst Hardy Plankton Recorder (LHPR) in the northeast Atlantic. Deep Sea Research Part I: Oceanographic Research Papers. 2004;51(9):1283–94. 10.1016/j.dsr.2004.04.002.

[122] Hélaouët P, Beaugrand G, Reygondeau G. Reliability of spatial and temporal patterns of C. finmarchicus inferred from the CPR survey. Journal of Marine Systems. 2016;153:18–24. 10.1016/j.jmarsys.2015.09.001.

[123] Pauly D, Hilborn R, Branch TA. Fisheries: Does catch reflect abundance? Nature. 2013;494(7437):303–6. 10.1038/494303a 23426308

[124] Conversi A, Fonda-Umani S, Peluso T, Molinero JC, Santojanni A, Edwards M. The Mediterranean Sea Regime Shift at the End of the 1980s, and Intriguing Parallelisms with Other European Basins. PLoS ONE. 2010;5(5):e10633 10.1371/journal.pone.0010633 20502704 PMC2873283

[125] Guyon I, Gunn S, Nikravesh M, Zadeh LA. Feature Extraction: Foundations and Applications: Springer Science & Business Media; 2006.

[126] Huthnance JM. Physical oceanography of the North Sea. Ocean and Shoreline Management. 1991;16(3–4):199–231. 10.1016/0951-8312(91)90005-M.

[127] Reygondeau G, Beaugrand G. Future climate-driven shifts in distribution of Calanus finmarchicus. Global Change Biology. 2011;17(2):756–66. 10.1111/j.1365-2486.2010.02310.x .

[128] Goberville E, Beaugrand G, Edwards M. Synchronous response of marine plankton ecosystems to climate in the Northeast Atlantic and the North Sea. Journal of Marine Systems. 2013;129:189–202.

[129] Reid PC, Beaugrand G. Global synchrony of an accelerating rise in sea surface temperature. Journal of the Marine Biological Association of the United Kingdom 2012;92:1435–50. 10.1017/S0025315412000549

[130] Head EJH, Melle W, Pepin P, Bagoien E, Broms C. On the ecology of Calanus finmarchicus in the Subarctic North Atlantic: A comparison of population dynamics and environmental conditions in areas of the Labrador Sea-Labrador/Newfoundland Shelf and Norwegian Sea Atlantic and Coastal Waters. Progress in Oceanography. 2013;114:46–63. 10.1016/j.pocean.2013.05.004 .

[131] Pierson JJ, Batchelder H, Saumweber W, Leising A, Runge J. The impact of increasing temperatures on dormancy duration in Calanus finmarchicus. Journal of Plankton Research. 2013;35(3):504–12. 10.1093/plankt/fbt022 .

[132] Conversi A, Peluso T, Fonda‐Umani S. Gulf of Trieste: a changing ecosystem. Journal of Geophysical Research: Oceans (1978–2012). 2009;114(C3).

[133] Sundby S. Recruitment of Atlantic cod stocks in relation to temperature and advection of copepod populations. Sarsia. 2000;85(4):277–98. .

[134] Beaugrand G, Kirby RR. Climate, plankton and cod. Global Change Biology. 2010;16(4):1268–80. 10.1111/j.1365-2486.2009.02063.x .

[135] McQuatters-Gollop A, Raitsos DE, Edwards M, Pradhan Y, Mee LD, Lavender SJ, et al A long-term chlorophyll data set reveals regime shift in North Sea phytoplankton biomass unconnected to nutrient trends. Limnology and Oceanography. 2007;52(2):635–48. .

[136] Llope M, Chan KS, Ciannelli L, Reid PC, Stige LC, Stenseth NC. Effects of environmental conditions on the seasonal distribution of phytoplankton biomass in the North Sea. Limnology and Oceanography. 2009;54(2):512–24. 10.4319/lo.2009.54.2.0512 .

[137] Edwards M, Reid P, Planque B. Long-term and regional variability of phytoplankton biomass in the Northeast Atlantic (1960–1995). ICES Journal of Marine Science: Journal du Conseil. 2001;58(1):39–49.

[138] Goberville E, Beaugrand G, Edwards M. Synchronous response of marine plankton ecosystems to climate in the Northeast Atlantic and the North Sea. Journal of Marine Systems. 2014;129:189–202.

[139] Schlüter MH, Kraberg A, Wiltshire KH. Long-term changes in the seasonality of selected diatoms related to grazers and environmental conditions. Journal of Sea Research. 2012;67(1):91–7.

[140] Utne KR, Hjollo SS, Huse G, Skogen M. Estimating the consumption of Calanus finmarchicus by planktivorous fish in the Norwegian Sea using a fully coupled 3D model system. Marine Biology Research. 2012;8(5–6):527–47. 10.1080/17451000.2011.642804 .

[141] Gardmark A, Casini M, Huss M, van Leeuwen A, Hjelm J, Persson L, et al Regime shifts in exploited marine food webs: detecting mechanisms underlying alternative stable states using size-structured community dynamics theory. Philosophical Transactions of the Royal Society B-Biological Sciences. 2015;370(1659). 10.1098/rstb.2013.0262 .

[142] Reubens JT, De Rijcke M, Degraer S, Vincx M. Diel variation in feeding and movement patterns of juvenile Atlantic cod at offshore wind farms. Journal of Sea Research. 2014;85:214–21. 10.1016/j.seares.2013.05.005.

