# Supplementary material for: A Novel, Unbiased Analysis Approach for Investigating Population Dynamics: A Case Study on Calanus finmarchicus and Its Decline in the North Sea
Source: PLoS One. 2016 Jul 1;11(7):e0158230. doi: 10.1371/journal.pone.0158230 (PMC4930201; doi:10.1371/journal.pone.0158230)
Supplement: S1 File — (PDF) [file pone.0158230.s001.pdf]

Supporting Information 1 for the paper:

A novel, unbiased analysis approach for investigating population dynamics: a case study on *Calanus finmarchicus* and its decline in the North Sea

Danny J. Papworth[1], Simone Marini[2], Alessandra Conversi[1,2]

[1] Faculty of Science and Technology, School of Marine Science and Engineering, University of Plymouth, Plymouth, Devon, United Kingdom, PL4 8AA.

[2] ISMAR Marine Sciences Institute in La Spezia, CNR National Research Council of Italy, Forte Santa Teresa, Loc. Pozzuolo, 19032 Lerici (SP), Italy.

1 Genetic Programming

Figure 1 summarizes the steps of the GP procedure used to approximate the target time-series.

Table 1 summarizes the parameters used in the GP procedure described in Figure 1. These parameters have been selected, after several experiments, in order to reduce the *overfitting* effects (i.e., resulting in a model that matches only the specific data set used and is not generalizable to other systems) and increase the generalization capability of the evolved functions.

Zooplankton abundance dynamics are still unknown and in this work we presume that they are not necessarily linear. For this reason the set of mathematical primitives shown in Equation (1) have been chosen for capturing a wide range of non-linearity degrees.

$$\mathcal{M} = \{+, -, *, /^*, sqrt^*, log^*, sin, cos, tan, atan\} \quad (1)$$

1. Randomly generate the initial parent population based on variables (time-series), mathematical operators, and constants;
2. Evaluate the fitness of each *individual* by instantiating its variables with the values of the corresponding time series;
3. Select two parent individuals for reproduction, according to their fitness; the individuals with higher fitness are assigned greater probability to mate;
4. Determine whether to apply the crossover to the two parents to reproduce offspring, or whether to clone one parent to the next generation; determine whether mutation occurs on the offspring individual;
5. Repeat the steps 3 and 4 until the predetermined population size is attained;
6. Use the offspring population as a new generation and return to step 2. This is iterated until the stop criterion is met;

Figure 1: The steps in the GP procedure used to evolve the functions that approximate the target time-series.

The operators $+, -, *, /^*$ can be used to evolve polynomial functions that provide good approximation capabilities mainly for simple time series. On the contrary, the operators $sqrt^*, log^*, sin, cos, tan, atan$ can be used to evolve functional forms capable to capture periodic trends and complex not linear behaviours.

The variables used for the generation of the initial population are those described in the paper, while the constants correspond to k real numbers randomly selected in the range $[-10, 10]$, where k is a natural number randomly selected in the range $[0, 10]$.

The initial population, based on the previously described mathematical operators, variables and constants, is generated according to the *ramped half-and-half technique* [2, 4]. In the settings applied in this work, each individual is represented by a tree whose maximum depth is 4. This value has been selected to reduce the complexity of the evolved individuals. The ramped half-and-half technique generates an initial population where half of the individuals are complete trees (i.e. all leaves are at the same depth) and the remaining individuals are not necessarily complete trees. This is done by using a range of depth limits in order to ensure that trees having a variety of sizes and shapes are generated.

The *raw fitness* of an individual corresponds to the approximation error between the individual (approximating function) and the target time series (here *Calanus finmarchicus*) and it is computed by the Root Mean Square Error

mathematical operators	$\mathcal{M}$
variables	shown in Table 1 of the main paper
constants	k randomly selected real numbers from the range $[-10, 10]$, where k is an integer number randomly selected in the range $[0, 10]$
initial population	ramped half-and-half
individual max depth	4
population size	1000
max generations	500
raw fitness	RMSE
scaled fitness	linear scaling
selector method	roulette wheel
crossover rate	0.9
mutation rate	0.0002
elitism	true
termination criterion	max generations $\vee$ raw fitness = 0.00

Table 1: The GP parameters used for the evolution of the functions approximating *C. finmarchicus* abundance.

(RMSE) defined in equation (2):

$$RMSE(O, A) \quad = \quad \sqrt{\frac{1}{N}\sum_{i=1}^{N}(x_i - x_i')^2}, \quad x_i \in O,\ x_i' \in A, \tag{2}$$

where N is the number of values in the *C. finmarchicus* time series O and in the approximating data A (biological, physical, and climate indices variables). The smaller the RMSE, the more the evolved individual approximates the observed time series.

The *scaled fitness* of an individual is a number expressed as a function of the raw fitness and represents how the individual fits relatively to the current population. The scaled fitness used in the proposed experiments is the linear scaling, where low raw fitness values (i.e. low RMSE) correspond to low scaled fitness values. The formal definition of the linear scaling can be found in [3].

the individuals are selected for reproduction depending on their fitness, following the *roulette wheel* strategy, according which the better an individual approximates the zooplankton time series (low scaled fitness), the higher is its probability to be selected.

In addition to the processes of selection, mating, crossover, and mutation, in each generation the best individual is cloned to the next generation (*elitism*). The elitism parameter increases the probability to obtain better approximating individuals generation after generation.

Finally the GP procedure ends when the termination criterion is met.

In the experiments proposed in this work, the initial population consists of 1000 individuals. The probability of crossover between two selected individuals

(parents) is 0.9. If crossover does not happen, only one of the two parents is randomly selected and cloned to the next generation. After the crossover or cloning, the mutation occurs with probability $2 \cdot 10^{-4}$. The termination criterion corresponds to the maximum number of generations allowed (i.e. 500), or when an evolved individual is evaluated with raw fitness equal to 0.

2 Relevance Analysis

A variable is deemed relevant if it appears in the population-pool more times than by chance. In order to identify the relevant variables, it is assumed that all the variables have the same probability to appear in the functions of the population-pool. A statistic test is used to accept or reject the previous assumption. Variables that violate the equal-probability assumption should be considered relevant.

The problem of identifying whether a variable $v \in \mathcal{V}$ is relevant or not, is brought back to a Bernoulli trial defined on the number of functions that contain v. The probability that v occurs in exactly k individuals of the population-pool is given by the binomial distribution shown in (3):

$$P(n,k,p) \quad = \quad \binom{n}{k} p^k \ (1-p)^{n-k}, \tag{3}$$

where n is the number of individuals of the population-pool obtained by the cross-validation and $p = \frac{1}{|\mathcal{V}|}$ is the probability that a variable appears in the functions of the population-pool. Within the proposed experiments, the parameters of the binomial distribution are: $n = 104$ and $p = \frac{1}{86} = 0.012$.

In the proposed experiments, the assumption that all the variables have the same probability to appear among the individuals of the population-pool is rejected with p-value equal to 0.001, as proposed in [1]. According to the proposed Bernoulli trial, this p-value corresponds to a variable occurrence greater than or equal to 7. This means that in our study all the variables whose occurrence among the individuals of the population-pool is greater than or equal to 7 are deemed relevant.

Figure 2 shows the binomial distribution where the variables whose occurrence is larger than the right red line are deemed relevant with p-value smaller than 0.001.

3 Modeling functions

Table 2 shows the abbreviations associated with the biological, physical and and climate indices variables and Table 3 shows the 19 modelling functions contained in the population-pool.

**Modelling Functions**	**Validation Error**
$atan(sin(Cod\_SSB)) - sin(spSST^2 \cdot H\_TAE \cdot Cod\_SSB)$	0.366
$cos(tan(H\_TAE)) \cdot sin(Cod\_SSB \cdot 3.366) \cdot (sin(tan(Cod\_SSB)))$	0.086
$cos(sin(cos(Cod\_age\_1))) - sin(atan(sin(PCI)))$	0.121
$atan(atan(\frac{wN\_Atl\_Net}{3.562})) \cdot \frac{1}{3.562 \cdot H\_TSB}$	0.123
$\frac{sin(Cod\_SSB)}{tan(cos(sin(smEng\_Chan\_E)))}$	0.199
$atan(cos(atan(9.747 \cdot H\_TAE)))$	0.108
$cos(sin(\sqrt{PCI})) - atan(sin(\sqrt{H\_TAE}))$	0.118
$\frac{cos(PCI)}{5.535 \cdot \sqrt{tan(H\_TAE)}}$	0.045
$\sqrt{cos(wSST)} - atan(\sqrt{H\_TAE})$	0.052
$cos(atan(\sqrt{PCI})) - atan(\sqrt{sin(H\_TSB)})$	0.0140
$\frac{sin(cos(1.384))}{PCI+H\_TSB}$	0.044
$log(H\_TSB) \cdot atan(atan(log(wSST)))$	0.061
$sin\left(\frac{cos(H\_TSB)}{cos(smEng\_Chan\_E\_flow)}\right) - sin(sin(PCI))$	0.022

$Cod\_SSB \cdot cos(atan(Cod\_SSB + PCI))$	0.026
$sin(cos(Cod\_SSB)) \cdot cos(PCI) \cdot atan(Cod\_SSB)$	0.026
$tan(cos(H\_TSB + Cod\_SSB)) \cdot sin(Cod\_SSB)$	0.036
$cos(H\_TSB + Cod\_SSB) \cdot sin(Cod\_SSB)$	0.015
$Cod\_SSB \cdot cos(cos(tan(smEng\_Chan\_E\_flow)))$	0.059
$atan\left(\frac{Cod\_SSB}{PCI}\right) - \left(tan(Cod\_SSB) \cdot atan(spSST)\right)$	0.064

Table 3: The 19 modelling-functions approximating *C. finmarchicus* abundance extracted from the population-pool (first column) and the corresponding validation error (second column). The prefixes *sp*, *sm* and *w*, represent the seasonal average of the variables and correspond respectively to springtime, summer and winter.

References

[1] Valen E. Johnson. Revised Standards for Statistical Evidence. *Proceedings of the National Academy of Sciences of the United States of America*, 110(48):19313–19317, November 2013.

[2] John R. Koza. *Genetic Programming: On the Programming of Computers by Means of Natural Selection.* MIT Press, Cambridge, MA, USA, 1992.

[3] Christian S. Perone. Pyevolve 0.6rc1. http://pyevolve.sourceforge.net/0_6rc1/.

[4] Riccardo Poli, William B. Langdon, and Nicholas Freitag McPhee. *A field guide to genetic programming.* `http://www.gp-field-guide.org.uk`, 2008.

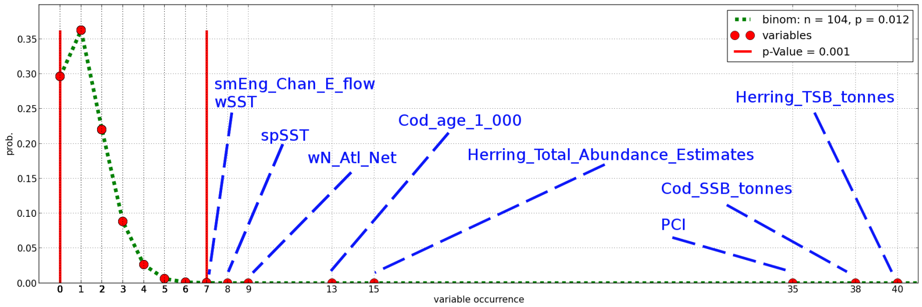

Figure 2: The probability mass function of binomial distribution (dotted green line) as defined in (3). The red dots represent the occurrence of each variable among the population-pool functions. The two vertical red lines correspond to left and right two-tails p-value equal to 0.001. Only the modelling variables are named into the diagram.

**Environmental variables**	**Short-Names**
North Atlantic Oscillation	NAO
East Atlantic Pattern	EA
East Atlantic West Russia Pattern	EAWR
Scandinavian Pattern	SCA
Polar Eurasia Pattern	POL
Atlantic Multidecadal Oscillation	AMO
Northern Hemisphere Temperature	NHT
North Atlantic Southward Flow	N_Atl_S
North Atlantic Northward Flow	N_Atl_N
North Atlantic Net Flow	N_Atl_Net
English Channel Eastward Flow	Eng_Chan_E
English Channel Westward Flow	Eng_Chan_W
English Channel Net Flow	Eng_Chan_Net
Sea Surface Temperature	SST
Salinity	Salinity
Total Nitrogen	T_Nit
Total Phosphorus	T_Phos
Silicate	SIL
Chlorophylla	Chl
Phytoplankton Colour Index	PCI
Chaetognaths Eyecount	Chaet
Total Fish Larvae	F_larvae
Herring (Claupea harengus) Total Stock Abundance	H_TAE
Herring (Claupea harengus) Total Stock Biomass	H_TSB
Cod (Gadus morhua) Aged 1 year	Cod_age_1
Cod (Gadus morhua) Spawning Stock Biomass	Cod_SSB

Table 2: The short-names (second column) associated to the environmental variables (first column).
