# Supplementary material for: A Novel, Unbiased Analysis Approach for Investigating Population Dynamics: A Case Study on Calanus finmarchicus and Its Decline in the North Sea
Source: PLoS One. 2016 Jul 1;11(7):e0158230. doi: 10.1371/journal.pone.0158230 (PMC4930201; doi:10.1371/journal.pone.0158230)
Supplement: S2 File — (PDF) [file pone.0158230.s002.pdf]

Supporting Information 2 for the paper:

A novel, unbiased analysis approach for investigating population dynamics: a case study on *Calanus finmarchicus* and its decline in the North Sea

Danny J Papworth[1], Simone Marini[2], Alessandra Conversi[1, 2*]

[1] Faculty of Science and Technology, School of Marine Science and Engineering, University of Plymouth, Plymouth, Devon, United Kingdom, PL4 8AA.

[2] ISMAR – Marine Sciences Institute in La Spezia, CNR – National Research Council of Italy, Forte Santa Teresa, Loc. Pozzuolo, 19032 Lerici (SP), Italy.

1. Approximation vs observation of *Calanus finmarchicus*

The GP procedure produced 104 approximating functions, of which 19 qualify as modelling functions, i.e., those containing just relevant variables (See S1). An example of a modelling functions ability to approximate the abundance of *C. finmarchicus* is presented in Figure 1 here. This modelling function was chosen because it contained only selected drivers, herring TSB and winter SST, and represents a synergistic relationship between a trophic and a physical driver. The function in Figure 1 reproduces the overall trend, but misses the specific peaks. This is possibly because the peaks depend on neither the herring TSB nor the winter SST.

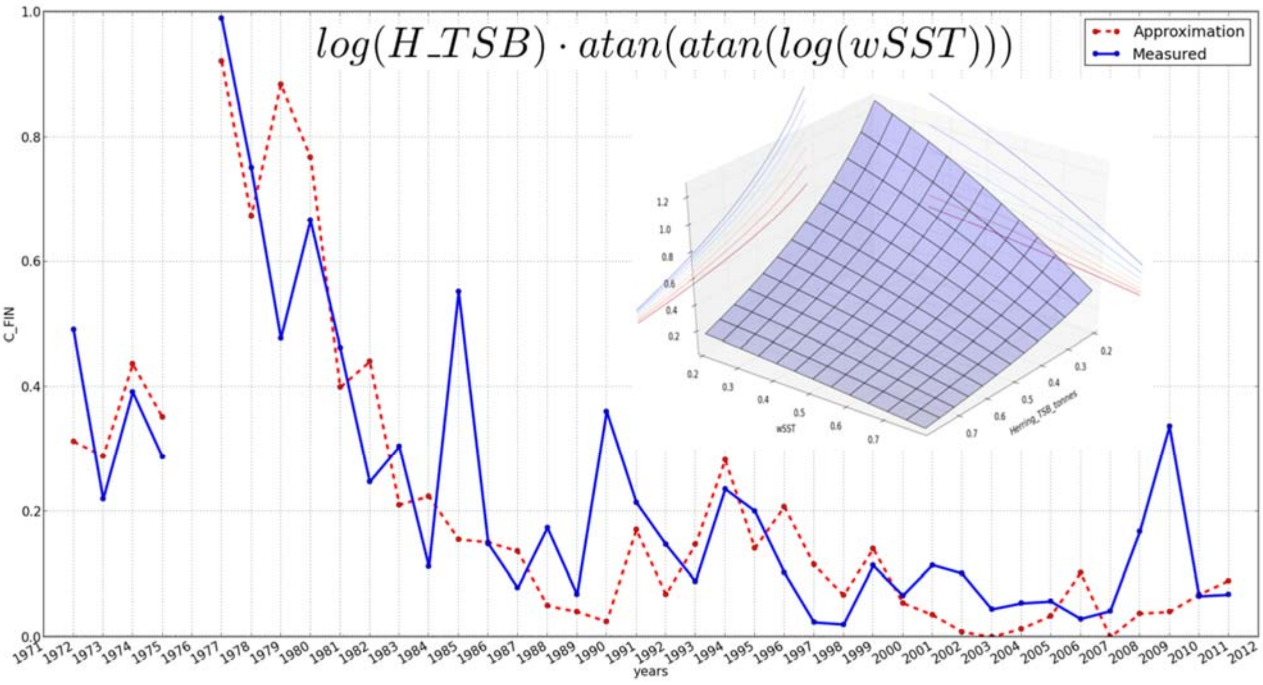

Fig 1: Example of a GP-produced modeling function. *Calanus finmarchicus* observed data (solid blue line) is shown together with the approximation (dotted red line) produced by the modelling function printed above the plot. The co-domain of the modelling function is depicted in the inset. This function has for input the herring total stock biomass and the winter sea surface temperature.

The accuracy of each modelling function in relation to the target variable is reported in S1, where a lower RMSE indicates a better prediction of the target value.

2. Cumulative Sums of the relevant variables

Cumulative sums analysis was conducted on *C. finmarchicus* to detect abrupt changes in the means of the 9 relevant variables identified by the GP-based analysis (Fig 2). The interpretation is based on the slope of the line on the chart: a constant deviation from the mean of the time series shows a constant slope. Persistent changes from the mean of the times series cause a persistent change of the slope [1]. A shift towards a lower mean abundance of *C. finmarchicus* was detected in 1986 (Fig 2A). This happened after shifts towards a higher mean in herring total abundance (1982) (Fig 2B)

and herring total stock biomass (1985) (Fig 2E), summer English Channel eastward flow (1984) (Fig 2J) and winter N. Atlantic net flow (1985) (Fig 2G). After the downward shift in *C. finmarchicus*, PCI saw an increase in 1987 (Fig 2D), while cod (both measured at age 1 and as spawning stock biomass) concurrently decreased also in 1987 (Fig 2C & F). Winter and spring SST shifted upwards in 1988 and 1989 respectively (Fig H & I).

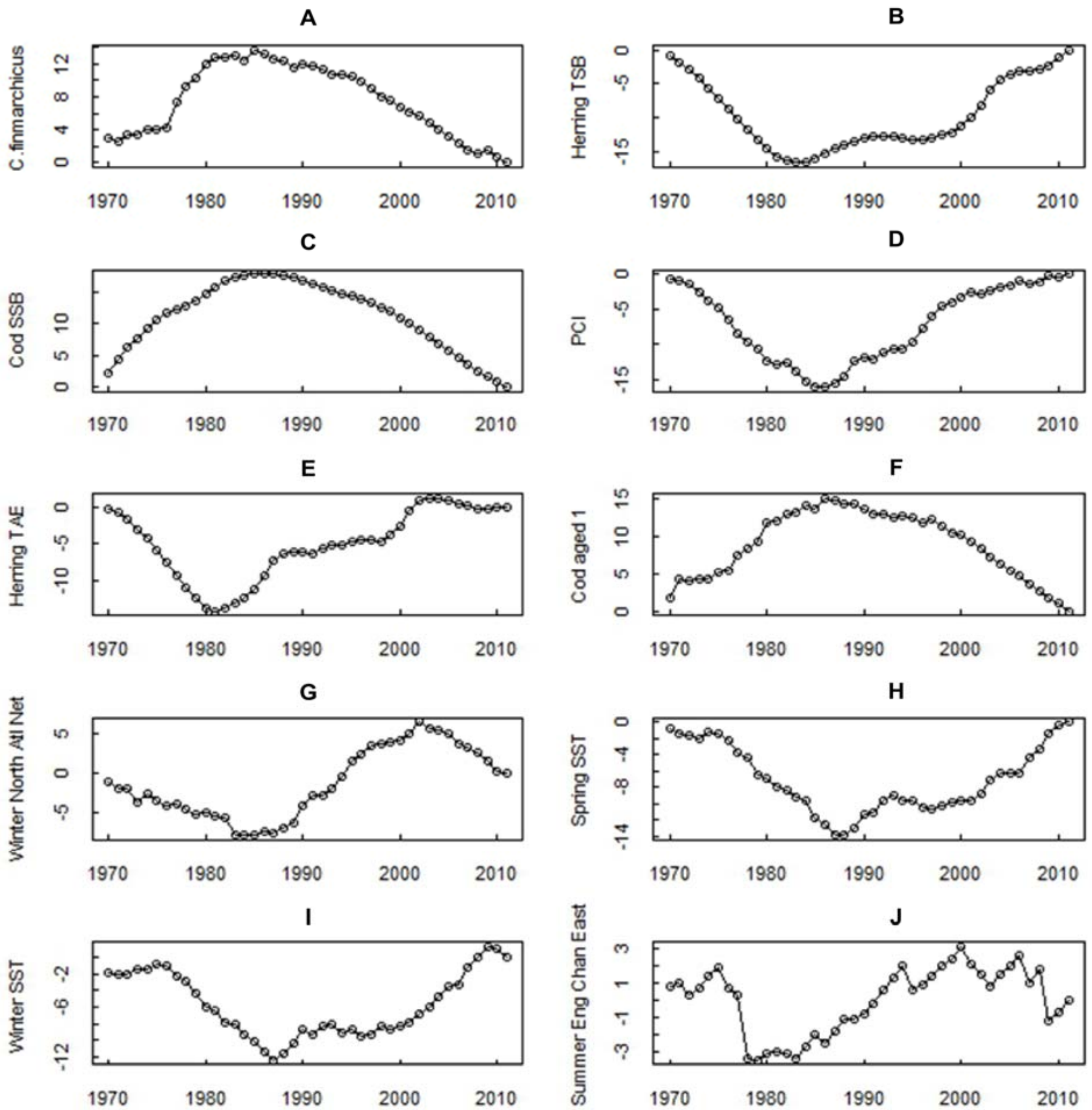

Figure 2: Cumulative Sum analysis of *C. finmarchicus* abundance and the 9 environmental variables identified. An abrupt, lasting change in the direction of the plotted line identifies a change point in the mean and therefore highlighting a potential regime shift in the time series.

References:

1. Ibanez F, Fromentin J, Castel J. Application of the cumulated function to the processing of chronological data in oceanography. Comptes Rendus De L Academie Des Sciences Serie Iii-Sciences De La Vie-Life Sciences. 1993;316(8):745-8.
