# Supplementary material for: A Novel, Unbiased Analysis Approach for Investigating Population Dynamics: A Case Study on Calanus finmarchicus and Its Decline in the North Sea
Source: PLoS One. 2016 Jul 1;11(7):e0158230. doi: 10.1371/journal.pone.0158230 (PMC4930201; doi:10.1371/journal.pone.0158230)
Supplement: S3 File — (DOCX) [file pone.0158230.s003.docx]

**Data availability information:**

Full monthly datasets for the North Atlantic Oscillation (NOA), East Atlantic Pattern (EA), East Atlantic West Russia Pattern (EAWR), Scandinavian Pattern (SCA) and Polar Eurasia Pattern (POL) can be found via the NOAA climate prediction centre:

<ftp://ftp.cpc.ncep.noaa.gov/wd52dg/data/indices/tele_index.nh>

Full monthly dataset for the Atlantic Multidecadal Oscillation (AMO) can be obtained from NOAA Earth System Research Laboratory Physical Sciences Division:

<http://www.esrl.noaa.gov/psd/data/timeseries/AMO/>

Full monthly dataset for Northern Hemisphere Temperature (NHT) can be obtained from NOAA National Centres for Environmental Information:

<http://www.ncdc.noaa.gov/monitoring-references/faq/anomalies.php#anomalies>

Monthly North Sea circulation data for inflow, outflow and net flow was obtained from **NORWegian ECOlogical Model System (NORWECOM)** through contact with Skogen, M. D.

Monthly salinity and sea surface temperature data was obtained from ICES surface data database:

<http://www.ices.dk/marine-data/dataset-collections/Pages/Ocean-surface-temperature.aspx>

Monthly data sets for nitrogen, phosphorus, silicate and chlorophyll-a concentrations were obtained from ICES bottle data database:

<http://ocean.ices.dk/HydChem/HydChem.aspx?plot=yes>

Plankton data sets; Phytoplankton colour index (PCI), Chaetognaths, fish larvae and Calanus finmarchicus, can be requested from Sir Alister Hardy Foundation for Ocean Science:

<http://www.sahfos.ac.uk/cpr-data/database.aspx>

Annual Herring stock abundance and stock biomass data was obtained from the ICES Working Group on Herring Assessment:

<http://www.ices.dk/community/groups/Pages/HAWG.aspx>

Annual cod aged 1 year and spawning stock biomass data was obtained from the ICES Working Group on the Assessment of Demersal Stocks in the North Sea and Skagerrak:

<http://www.ices.dk/community/groups/Pages/WGNSSK.aspx>
